# Interfacial percolation enables high-conductivity lean-lithium oxychloride electrolytes for all-solid-state batteries

**DOI:** 10.1093/nsr/nwag411

**Published:** 2026-07-03

**Authors:** Deyuan Li, Guangwen Zhang, Boyuan Xu, Feifei Luo, Kongying Zhu, Guohong Liang, Tianran Yan, Weiqi Hou, Liang Zhang, Qisheng Wu, Chunpeng Yang

**Affiliations:** Tianjin Key Laboratory of Advanced Carbon and Electrochemical Energy Storage, School of Chemical Engineering and Technology, Tianjin University, Tianjin 300350, China; Tianjin Key Laboratory of Advanced Carbon and Electrochemical Energy Storage, School of Chemical Engineering and Technology, Tianjin University, Tianjin 300350, China; Division of Energy & Environmental Materials, Suzhou Laboratory, Suzhou 215123, China; Tianjin Key Laboratory of Advanced Carbon and Electrochemical Energy Storage, School of Chemical Engineering and Technology, Tianjin University, Tianjin 300350, China; Analysis and Test Center, Tianjin University, Tianjin 300350, China; Tianjin Key Laboratory of Advanced Carbon and Electrochemical Energy Storage, School of Chemical Engineering and Technology, Tianjin University, Tianjin 300350, China; Institute of Functional Nano & Soft Materials, Jiangsu Key Laboratory of Advanced Negative Carbon Technologies, Soochow University, Suzhou 215123, China; Tianjin Key Laboratory of Advanced Carbon and Electrochemical Energy Storage, School of Chemical Engineering and Technology, Tianjin University, Tianjin 300350, China; Institute of Functional Nano & Soft Materials, Jiangsu Key Laboratory of Advanced Negative Carbon Technologies, Soochow University, Suzhou 215123, China; Division of Energy & Environmental Materials, Suzhou Laboratory, Suzhou 215123, China; Tianjin Key Laboratory of Advanced Carbon and Electrochemical Energy Storage, School of Chemical Engineering and Technology, Tianjin University, Tianjin 300350, China

**Keywords:** all-solid-state batteries, solid-state electrolytes, interfacial percolation, lithium oxychloride electrolytes, lean-lithium electrolytes

## Abstract

Inorganic solid-state electrolytes (SSEs) underpin the development of all-solid-state batteries (ASSBs), but their ionic conductivity relies heavily on Li-concentrated or even Li-stuffed materials, which not only elevates Li consumption but also compromises their (electro)chemical stability. Here, we establish an interfacial percolation strategy that enables high ionic conductivity at lean-Li content, breaks the conventional dependence of ionic conductivity on high Li content and allows tuning of electrolyte properties such as high-voltage stability. With a typical percolative SSE that consists of Ta_2_O_5_ nanocrystals embedded in an amorphous matrix (Ta_2_O_5_*·a*-LTOC), we demonstrate that, contrary to common perception, insulating dispersoids can lower Li concentration while increasing ionic conductivity by forming an interfacial percolation network. Ta_2_O_5_*·a*-LTOC achieves a high ionic conductivity of 15.2 mS cm^−1^ at a Li content of 1.46 wt% (6.4 mol L^−1^), together with high-voltage stability up to 4.8 V. As a result, ASSBs using Ta_2_O_5_·*a*-LTOC and high-nickel cathode materials deliver a long cycle life of 5000 cycles at 2 C, stable high-rate cycling of 4000 cycles at 6 C and high-loading performance with an areal capacity >3 mAh cm^−2^ and 80.4% capacity retention over 800 cycles. This interfacial percolation strategy provides a universal design route to lean Li, highly conductive SSEs with tunable properties, thereby enabling high-energy and fast-charging ASSBs.

## Teaser

Interfacial percolation enables lean-lithium solid electrolytes with high ionic conductivity and high-voltage stability, providing a universal design strategy for high-energy and fast-charging ASSBs.

## INTRODUCTION

Inorganic solid-state electrolytes (SSEs) are expected to revolutionize liquid electrolytes and drive the advancement of all-solid-state batteries (ASSBs) as next-generation energy storage systems [1–5]. Significant progress has been made in SSEs, particularly in improving their ionic conductivity, which is critical for the electrochemical performances of ASSBs [6–11]. Generally, the Li ionic conductivity of SSE is improved by increasing Li content/concentration or even using Li-stuffed materials, as the Li ionic conductivity (*σ*) is ideally determined by *σ* = *qnμ*, where *q* is the product of charge, *n* is carrier concentration and *μ* is mobility of carriers [12–15]. Taking garnet-type SSEs (Li_α_La_3_M_2_O_12_, M denotes various cations) as an example, it is found that as Li content increases from 3 to 7 (41.3 mol L^−1^ in Li_7_La_3_Zr_2_O_12_), the ionic conductivity of garnet increases almost exponentially with Li content [16]. Similarly, increasing Li content (up to ∼40 mol L^−1^ in argyrodites) can also significantly enhance ionic conductivity for sulfide SSEs [13–15]. However, such a high Li concentration of SSEs not only relies on the limited Li resources [17], but also decreases the stability of SSEs due to the high activity of the stuffing Li ions. Thus, it is imperative to develop strategies that reduce Li content while maintaining high ionic conductivity and ensuring comprehensive performance (e.g. electrochemical stability, deformability and air stability) for SSEs.

In fact, in Li-stuffed SSEs, not all Li ions contribute to mobile charge carriers, as some Li ions are dedicated to maintaining the structure of SSEs [12,15,16,18]. In traditional inorganic SSEs, Li ion conduction occurs only when the framework sites are adequately filled with structural Li ions [19], which increases the Li content and even potentially impedes ionic mobility (*μ*). Amorphous SSEs can reduce the reliance on Li ions that are bound within the lattice framework. Introducing Li-free insulating phases is a straightforward strategy to reduce the Li content in SSE systems; however, such an approach is generally expected to decrease the ionic conductivity. In contrast, percolation theory suggests that rational introduction of insulating dispersoid phases into a conductive matrix can instead increase the overall conductivity upon forming a percolation network [20–22]. Thus, percolation theory offers a promising route to solve the contradiction between lean-Li content and high conductivity for SSEs.

In this work, we establish an interfacial percolation strategy for inorganic SSEs, using a representative percolation oxychloride solid electrolyte (Li_1.6_Ta_2.4_O_3_Cl_7.6_, denoted as Ta_2_O_5_·*a*-LTOC) composed of a conductive amorphous matrix and Ta_2_O_5_ dispersoids to achieve lean-Li content, high ionic conductivity and high-voltage stability. Experimental characterizations demonstrate that the formation of an interphase between the amorphous matrix and Ta_2_O_5_ dispersoids induces Li-ion redistribution and enables efficient ionic transport through a continuous interfacial percolation network. Benefiting from the interfacial percolation mechanism, these percolative SSEs achieve high ionic conductivity (up to 15.2 mS cm^−1^ in Ta_2_O_5_·*a*-LTOC with a Li content of 1.46 wt% or 6.4 mol L^−1^), exceptionally low Li content (as low as 0.48 wt% in Li_0.5_Ta_2.4_O_3_Cl_6.5_, <1/10 of previously reported inorganic SSEs) and high-voltage stability (4.8 V vs Li^+^/Li). Due to the high conductivity and high-potential stability of the Ta_2_O_5_·*a*-LTOC, ASSBs with LiNi_0.9_Co_0.05_Mn_0.05_O_2_ (Ni90) cathode demonstrate long cycle life at high rates, achieving 5000 cycles at 2 C and 4000 cycles at 6 C. This interfacial percolation design provides a universal strategy for inorganic SSEs that is fundamentally different from the conventional Li-enrichment route, enabling high ionic conductivity at lean-Li content together with tunable properties, such as high-voltage stability, for advanced ASSBs.

## RESULTS AND DISCUSSION

### Construction of percolative SSEs

**Figure 1.**
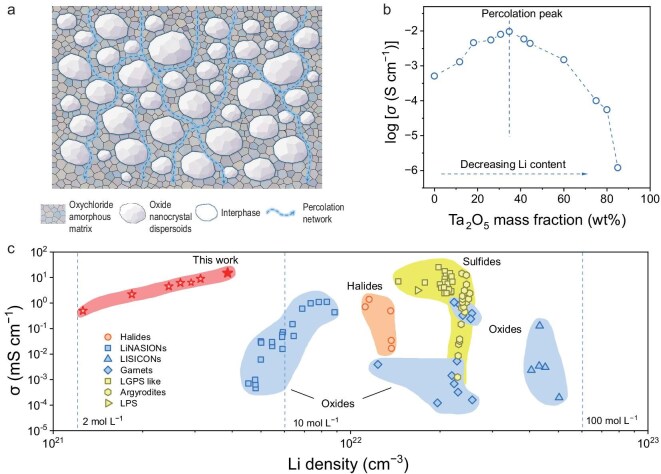
Ion percolation mechanism and Li^+^ conductivity versus Li density in different SSEs. (a) Schematic diagram of percolative SSEs with the transport mechanism. (b) Ionic conductivity as a function of Ta_2_O_5_ mass fraction for SSEs prepared from 1.63 LiCl:1.2 TaCl_5_:*k* Ta_2_O_5_ (Fig. S1 and Table S1). (c) Comparison of Li density and ionic conductivity for various types of electrolytes [17].

To validate the interfacial percolation strategy, we constructed percolative SSEs by introducing Li-free oxide dispersoids into an amorphous matrix (Fig. 1a). The ionic conductivities of resulting SSEs (Li*_i_*Ta*_j__+__2k_*O*_5k_*Cl*_i__+__5j_*), synthesized by ball milling a mixture of LiCl:TaCl_5_:Ta_2_O_5_ with a molar ratio of *i*:*j*:*k*, exhibit a non-monotonic dependence on composition (Fig. S1a). With increasing estimated amorphous phase proportion (*ϕ*_a_), effective ionic conduction starts to emerge at ∼15 wt%, which is considered the effective percolation threshold ($\phi_c^{\rm eff}$) (Fig. S1b). Moreover, the conductivity-proportion evolution shows characteristic percolation behavior and follows the percolation scaling law (Fig. S1) [20,23], supporting the transition from isolated amorphous/interfacial regions to connected Li^+^ transport pathways [20,24]. The ionic conductivity reaches a maximum at 34.7 wt% Ta_2_O_5_ for Li_1.63_Ta_2.4_O_3_Cl_7.63_ (Fig. 1b), indicating the formation of an optimized amorphous/interfacial transport network. Guided by this percolation behavior, we further optimized the composition and obtained the representative percolation oxychloride solid electrolyte, Li_1.6_Ta_2.4_O_3_Cl_7.6_ (denoted as Ta_2_O_5_·*a*-LTOC), which delivers an ionic conductivity of 15.2 mS cm^−1^ at a lean-Li content of 1.46 wt% (6.4 mol L^−1^) (Fig. S2 and Table S1). Notably, compared to the oxide, halide and sulfide SSEs reported in the literature, our percolative SSEs solve the contradiction between the lean-Li content and high conductivity, reaching ionic conductivities up to 15.2 mS cm^–1^, while reducing Li content to as low as 0.48 wt% (2 mol L^–1^ in Li_0.5_Ta_2.4_O_3_Cl_6.5_), among inorganic SSEs reported to date (Fig. 1c).

### Structure analysis of percolative SSEs

**Figure 2.**
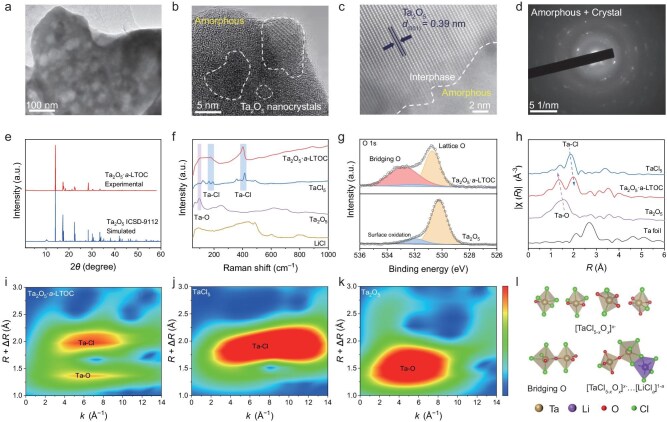
Percolation and local structure analysis. (a) Cryo-TEM, (b) high-resolution TEM, (c) HAADF-STEM and (d) SAED pattern images of Ta_2_O_5_·*a*-LTOC. (e) SXRD pattern, (f) Raman spectra, (g) XPS spectra and (h) FT-EXAFS spectra of Ta_2_O_5_·*a*-LTOC and commercial Ta_2_O_5_, TaCl_5_ or LiCl as labeled. Wavelet-transformed EXAFS contour plots of (i) Ta_2_O_5_·*a*-LTOC, (j) TaCl_5_ and (k) Ta_2_O_5_ at Ta *L*_3_-edge with a *k*^3^ weighting. (l) Schematic illustrations of the main possible structural configurations of [TaCl_5−_*_x_*O*_x_*]*^x^*^−^ and [TaCl_5−_*_x_*O*_x_*]*^x^*^−^···[LiCl*_a_*]^1−^*^a^*.

We employed cryogenic transmission electron microscope (cryo-TEM) to investigate the microstructure, composition and elemental distribution of Ta_2_O_5_·*a*-LTOC. Figure 2a shows that Ta_2_O_5_·*a*-LTOC comprises a matrix and randomly dispersed nanoparticles, a typical percolation structure [20,24]. High-resolution TEM image (Fig. 2b) shows that Ta_2_O_5_ dispersoids are embedded in the amorphous matrix and surrounded by an interphase with disorder or defects, which could induce space-charge zones, local defects and chemical-potential gradients for Li^+^ redistribution [25–28]. Atomic resolution high-angle annular dark-field scanning TEM (HAADF-STEM) image (Fig. 2c) further confirms the effective embedding of Ta_2_O_5_ nanocrystals within the amorphous matrix and the formation of an interphase at the atomic scale. Energy dispersive X-ray spectroscopy (EDS) mapping analysis (Fig. S3) discloses that in Ta_2_O_5_·*a*-LTOC, O is uniformly distributed throughout the particle, indicating that O participated in the reaction between LiCl and TaCl_5_ to form the amorphous Li-Ta-O-Cl matrix. Selected area electron diffraction (SAED, Fig. 2d) and powder X-ray diffraction (XRD) analyses (Figs S4 and S5) reveal that Ta_2_O_5_·*a*-LTOC consists of an amorphous phase and a crystalline Ta_2_O_5_ phase (ICDD 00-25-0922). High-resolution synchrotron-based XRD (SXRD) analysis (Fig. 2e and Fig. S6) shows that the crystalline phase in Ta_2_O_5_·*a*-LTOC is Ta_2_O_5_ crystal (ICSD-9112) without other crystalline impure phases (e.g. LiCl and Li_2_O). These results demonstrate that Ta_2_O_5_·*a*-LTOC comprises an amorphous matrix with embedded Ta_2_O_5_ nanocrystals, forming a percolation structure with Ta_2_O_5_-derived interfacial regions.

To gain a more comprehensive understanding of the bonding structure of Ta_2_O_5_·*a*-LTOC, we conducted a series of in-depth analyses. Raman spectrum of Ta_2_O_5_·*a*-LTOC shows Ta–Cl-related vibrational features at around 176 and 401 cm^−1^ (Fig. 2f), which are broadened and less resolved compared with the TaCl_5_ reference, supporting substantial modification of local Ta–Cl bonding environments during high-energy ball milling, likely associated with the disruption/rearrangement of chloride-containing Ta units and the evolution of a disordered oxychloride structure [29–32]. The Ta–O-related features in Ta_2_O_5_·*a*-LTOC (Fig. 2f) become broad and less distinguishable from those of Ta_2_O_5_, suggesting oxygen-containing Ta local environments with reaction-induced disorder and partial involvement of Ta_2_O_5_-derived O species in the oxychloride framework, accompanied by defects, local structural heterogeneity and amorphization [31–34]. The X-ray photoelectron spectroscopy (XPS) results (Fig. 2g and Fig. S7) and Fourier-transform infrared spectroscopy (FT-IR) spectrum (Fig. S8) also support the presence of bridging O in Ta_2_O_5_·*a*-LTOC, which is consistent with the previous reports that bridging O serves as joints (Ta-O-Ta) in [TaCl_5−_*_x_*O*_x_*]*^x^*^−^ coordination [31]. The bridging O in Ta_2_O_5_·*a*-LTOC induces a wide range of distortions in Li sites.

X-ray absorption spectroscopy measurements were conducted to investigate the local structure of Ta_2_O_5_·*a*-LTOC. The normalized X-ray absorption near-edge structure (XANES) spectra (Fig. S9) of Ta_2_O_5_·*a*-LTOC at the Ta-*L*_3_ edge show a decreased intensity and broadening of the white line compared to TaCl_5_ and Ta_2_O_5_, which could be attributed to the presence of an amorphous phase, disorder or defects in Ta_2_O_5_·*a*-LTOC [35]. Ta *L*_3_-edge XANES linear combination fitting (Fig. S10) gives an estimated Ta_2_O_5_-like contribution of 32.4 wt% (originally 34.76 wt%), indicating that most Ta_2_O_5_ remains as oxidelike dispersoids while a fraction participates in forming the amorphous oxychloride phase. Fourier-transform extended X-ray absorption fine structure (FT-EXAFS) results (Fig. 2h) reveal coexisting Ta–O and Ta–Cl scattering contributions in Ta_2_O_5_·*a*-LTOC, suggesting the reconstruction of the Ta local coordination environment. Additionally, wavelet-transformed EXAFS (Fig. 2i–k) shows decreased intensities of Ta–O and Ta–Cl scattering features in Ta_2_O_5_·*a*-LTOC, further supporting the rearrangement of Ta–O/Ta–Cl local environments. The fitted EXAFS results (Fig. S9 and Table S2) show that the Ta–O, Ta–Cl, Ta–O–Ta and Ta–Ta paths exhibit coordination parameters distinct from those of crystalline Ta_2_O_5_ and TaCl_5_ references. The shortened Ta–O distances, broadened Ta–Cl bond-length distribution and shifted Ta–O–Ta/Ta–Ta higher-shell correlations indicate that part of O from Ta_2_O_5_ contributes to the amorphous Li–Ta–O–Cl structure and reconstructs the Ta local coordination environment, possibly forming Ta-centered mixed oxychloride units described as [TaCl_5−_*_x_*O*_x_*]*^x^^−^* (*x* < 5) with Ta–O–Ta linkages (as illustrated in Fig. 2l). The combined Raman, EDS, TEM, XRD and SXRD analyses indicate the participation of O in the interfacial reaction and the formation of oxygen-containing Ta local environments in the amorphous oxychloride phase. In the [TaCl_5−_*_x_*O*_x_*]*^x^^−^* (*x* < 5) based mixed oxychloride framework, terminal Cl ligands weakly interact with Li ions, while O incorporation and structural disorder distort the Li-ion migration pathways, thereby facilitating low-barrier Li-ion hopping (Fig. 2l and Fig. S7a) [31]. These structural analyses confirm that Ta_2_O_5_ nanocrystals are effectively embedded in the amorphous matrix to form a percolation structure, while Ta_2_O_5_ also acts as functional oxide dispersoids that participate in interfacial and amorphous-matrix reconstruction.

### Electrochemical properties and ion transport mechanism

**Figure 3.**
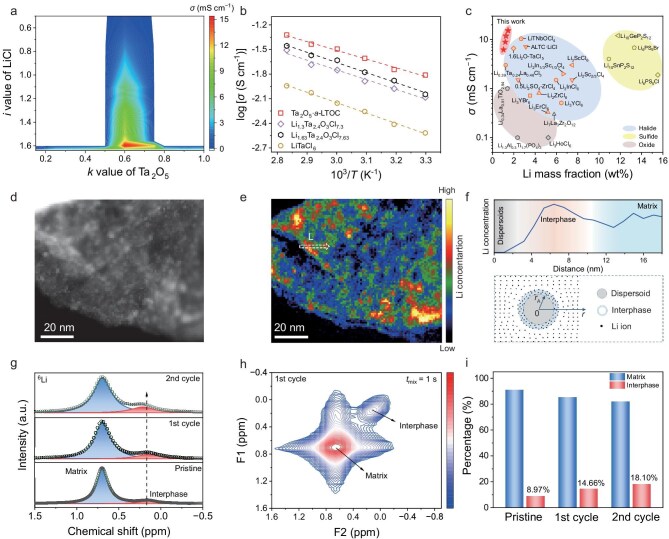
Electrochemical properties and ionic percolation analysis. (a) Ionic conductivities of *i* LiCl:1.2 TaCl_5_:*k* Ta_2_O_5_ (molar ratio) compositions. (b) Temperature-dependent ionic conductivities of various SSEs. (c) Comparison of ionic conductivity as a function of Li mass fraction among typical oxide, sulfide and halide SSEs. (d) STEM image where the (e) Li-*K*_edge_ SR-EELS mapping was acquired, and (f) corresponding Li concentration profile with a schematic of microgradients of Li concentration at the dispersoid interphase. (g) 1D ^6^Li MAS NMR spectra for Ta_2_O_5_·*a*-LTOC at different cycling states. (h) 2D ^6^Li EXSY spectrum of Ta_2_O_5_·*a*-LTOC after the first cycle. (i) 1D ^6^Li MAS NMR quantification results for Ta_2_O_5_·*a*-LTOC at different cycling states.

We tested ionic conductivities of the percolative SSEs with various proportions of *i* LiCl, *j* TaCl_5_ and *k* Ta_2_O_5_ (Fig. 3a, Fig. S2 and Table S1). The introduction of Ta_2_O_5_ significantly enhances ionic conductivity, reduces Li content and improves synthesis efficiency compared with Li_4_Ta_3_Cl_19_ and LiTaCl_6_ (Fig. S11). This comparison indicates that the high conductivity of Ta_2_O_5_·*a*-LTOC is closely related to the Ta_2_O_5_-derived dispersoid/interphase network, rather than the matrix contribution alone. We achieved a low Li content of 2 mol L^−1^ (0.48 wt%) in Li_0.5_Ta_2.4_O_3_Cl_6.5_ with an ionic conductivity of 0.5 mS cm^−1^, and 15.2 mS cm^−1^ in Ta_2_O_5_·*a*-LTOC with a Li content of 1.46 wt% (6.4 mol L^−1^). Arrhenius activation energies (*E*_a_) of the SSEs were determined based on the temperature-dependent ionic conductivities obtained by the electrochemical impedance spectroscopy. *E*_a_ of Ta_2_O_5_·*a*-LTOC (Li_1.6_Ta_2.4_O_3_Cl_7.6_), Li_1.3_Ta_2.4_O_3_Cl_7.3_, Li_1.63_Ta_2.4_O_3_Cl_7.63_ and LiTaCl_6_ are 0.24, 0.26, 0.27 and 0.28 eV, respectively (Fig. 3b and Fig. S12). The conductivity variation cannot be explained by *E*_a_ alone, although the small *E*_a_ difference still affects the Arrhenius exponential term. Additional analyses of pre-exponential factor (*σ*_0_), *σ*/*n*_Li,wt_ and room-temperature ^7^Li magic-angle spinning nuclear magnetic resonance (MAS NMR) T_1_ and T_2_ relaxation behavior indicate that ionic transport is governed by coupled effects of *E*_a_, prefactor contribution, Li-content utilization, local Li relaxation and interfacial pathway connectivity (Fig. S13 and Table S3). Figure S14 shows that Ta_2_O_5_·*a*-LTOC exhibits a low electronic conductivity of 4.2 × 10^−10^ S cm^−1^, confirming its suitability as a Li-ion conductor for batteries. Compared with most reported halide, sulfide and oxide SSEs (Fig. 3c and Table S4), Ta_2_O_5_·*a*-LTOC delivers a high ionic conductivity and a low Li mass fraction owing to its percolation structure.

To understand how the Ta_2_O_5_ dispersoids improve Li^+^ percolation and conduction, we performed spatially resolved electron energy loss spectroscopy (SR-EELS) to analyze the Li concentration distribution in Ta_2_O_5_·*a*-LTOC. Figure 3d shows the STEM image of Ta_2_O_5_·*a*-LTOC with dispersoids (bright spots, Fig. S15) embedded in the matrix (amorphous Li-Ta-O-Cl). SR-EELS mapping results (Fig. 3e) reveal that the Li distribution is non-uniform but forms a continuous high-concentration pathway along the dispersoid interphase, thereby establishing a continuous ion transport network in Ta_2_O_5_·*a*-LTOC. Due to space-charge-layer effects, chemical-potential redistribution, defects and chemically reconstructed local coordination environments, a microgradient Li concentration peak forms at the interphase region (Fig. 3f), playing a crucial role in constructing interconnected ion transport networks, facilitating ion transport and significantly enhancing ionic conductivity [21,24,27,28,36]. In contrast, Li_1.63_Ta_1.5_O_0.75_Cl_7.63_ with a low Ta_2_O_5_ dispersoid content (Fig. S16) exhibits limited interfacial regions and cannot form continuous Li^+^ percolation networks, resulting in its relatively low ionic conductivity. Moreover, thermal-treatment control verifies the essential role of the amorphous matrix/interphase in maintaining the connected interfacial percolation structure and efficient Li^+^ transport (Fig. S17). The observed Li distribution in Ta_2_O_5_·*a*-LTOC directly supports an interfacial percolation mechanism, in which the dispersoid/matrix interphase forms a continuous ion-conduction network.

To demonstrate the Li ion percolation mechanism in Ta_2_O_5_·*a*-LTOC, we performed ^6^Li isotopic tracing exchange measurements using MAS NMR. ^6^Li NMR result (Fig. 3g and Fig. S18) reveals that the Ta_2_O_5_*·a-*LTOC exhibits two Li resonances at 0.70 ppm (91.03%) and 0.17 ppm (8.97%), corresponding to the coordination Li in amorphous matrix (Li[Cl_6_]^−^) and interfacial Li at the interphase (Li[Cl_6-_*_x_*O*_x_*]^−^) around the Ta_2_O_5_ nanocrystals, respectively. Symmetric cells using ^6^Li were assembled to determine Li coordination changes at different cycling times. During the cycling process, ^6^Li^+^ ions (from ^6^Li alloy electrodes with 95% ^6^Li in Li metal) migrate through the electrolyte, replacing the Li ions in Ta_2_O_5_·*a*-LTOC (with naturally 7.5% ^6^Li and 92.5% ^7^Li) by ^6^Li. The Li^+^ transport channels are thus traced by the variations of ^6^Li in the cycled electrolytes with high-resolution Li isotope solid-state NMR. A 2D exchange spectroscopy (EXSY) experiment was conducted to probe the spontaneous ^6^Li-ion exchange of different Li ion environments. In the 2D contour plot (Fig. 3h), two circular regions on the diagonal represent the two Li environments of amorphous matrix and interphase, respectively. Off-diagonal peaks appear at mixing time *t*_mix_ = 1 s (Fig. 3h), demonstrating the significant exchange of Li^+^ ions between the matrix and the interphase. Moreover, the fitting results of the 1D ^6^Li NMR spectra (Fig. 3g and i) at different cycling states show that, while Li mainly distributes in the matrix, the ^6^Li content in the interphase increases from 8.97% to 14.66% after the first cycle, and further to 18.10% after the second cycle. The increase of ^6^Li-ion concentration in the interphase demonstrates that Li^+^ ions migrate preferentially through the interphase, indicating that the interphase region contributes significantly to ion conduction. The results above collectively confirm that Ta_2_O_5_·*a*-LTOC aligns with the percolation model, forming a high-rate, interconnected percolating ion transport network along the interphase region, fundamentally different from bulk-phase ion conduction. The high ionic conductivity of Ta_2_O_5_·*a*-LTOC at lean-Li content is attributed to the interfacial percolation network and the synergistic effects of defects, the amorphous matrix and the optimization of the percolation structure, which together ensure efficient ion transport.

### Electrochemical performance of ASSBs

**Figure 4.**
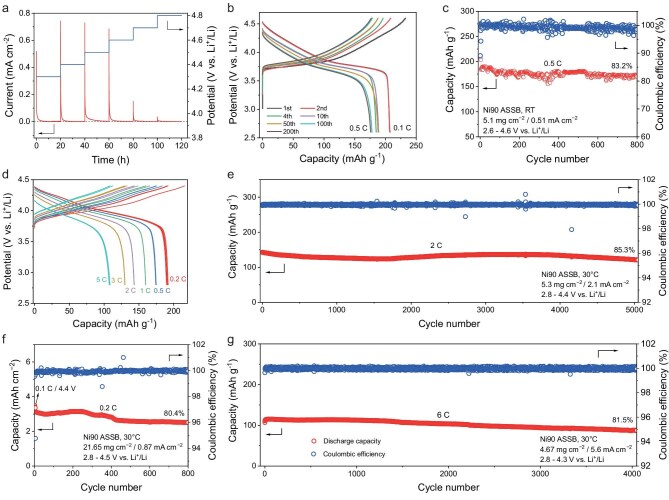
Electrochemical performance of ASSBs with Ta_2_O_5_·*a*-LTOC. (a) EFT of LiNi_0.9_Co_0.05_Mn_0.05_O_2_ (Ni90) ASSB with Ta_2_O_5_·*a*-LTOC. (b) High-voltage charge–discharge curves and (c) corresponding cycling performance of Ni90 ASSB with Ta_2_O_5_·*a*-LTOC at 0.5 C in a voltage range of 2.6–4.6 V versus Li^+^/Li. (d) Charge–discharge curves of Ni90 ASSBs with Ta_2_O_5_·*a*-LTOC at different rates. (e) Cycling performance of Ni90 ASSBs with Ta_2_O_5_·*a*-LTOC at 2 C in a voltage range of 2.8–4.4 V versus Li^+^/Li at 30°C. (f) High-loading cycling performance of Ni90 ASSB with Ta_2_O_5_·*a*-LTOC in a voltage range of 2.8–4.5 V versus Li^+^/Li at 30°C. (g) High-rate cycling performance of Ni90 ASSBs with Ta_2_O_5_·*a*-LTOC at 6 C in a voltage range of 2.8–4.3 V versus Li^+^/Li at 30°C.

We systematically evaluated the electrochemical performance of Ta_2_O_5_·*a*-LTOC in ASSBs with Ni90 cathode. The linear sweep voltammetry (LSV) result shows that the oxidative onset of Ta_2_O_5_*·a-*LTOC occurs near 4.5 V versus Li^+^/Li (Fig. S19). However, the electrochemical stability window obtained from LSV does not necessarily represent the practical operating voltage of ASSBs, because interphase layers could form at the electrolyte–electrode interface [1,37,38]. To further assess the practical high-voltage tolerance under full-cell conditions, we performed an electrochemical floating test (EFT) using Ni90 ASSBs with Ta_2_O_5_·*a*-LTOC. The cell was first charged to 4.3 V at 0.1 C, followed by stepwise voltage holds from 4.3 to 4.8 V for 20 h at each step to monitor leakage current associated with electrolyte degradation. Ta_2_O_5_·*a*-LTOC based ASSB shows low residual leakage current during voltage holds up to 4.8 V (Fig. 4a), indicating good high-voltage tolerance in practical ASSBs [39,40]. The interfacial evolution under the stringent 4.8 V condition was further examined by *in situ* distribution of relaxation times (DRT) and post-cycling scanning Electron Microscopy (SEM)/XPS analyses. *In situ* DRT analyses show stable impedance evolution during the initial high-voltage charge–discharge process up to 4.8 V, with no pronounced irreversible impedance buildup (Figs S20 and S21). Post-cycling SEM shows no discernible byproducts after two cycles at 4.8 V, whereas XPS detects trace oxidized chloride species (Fig. S22), indicating that limited interfacial oxidation occurs and that long-term operation at 4.8 V remains challenging.

We then evaluated the cycling performance of Ni90 ASSBs with Ta_2_O_5_·*a*-LTOC. When charged to 4.6 V versus Li^+^/Li, the Ni90 ASSB with Ta_2_O_5_·*a*-LTOC remains stable for 800 cycles at 0.5 C with a capacity retention of 83.2% (Fig. 4b and c). Even with an increased upper cutoff voltage of 4.8 V, the Ni90 ASSB retains 83.4% of its capacity after 300 cycles at 3 C (Fig. S23), further supporting the practical high-voltage tolerance of Ta_2_O_5_·*a*-LTOC.

The high ionic conductivity and high electrochemical stability of Ta_2_O_5_·*a*-LTOC are expected to facilitate Li^+^ transport and stable electrode/electrolyte interfaces in practical ASSBs [41,42]. Galvanostatic intermittent titration (GITT) measurements were performed to estimate the Li⁺ diffusion coefficient (*D*_Li_) and the overpotential during charge/discharge. The GITT result shows a high Li⁺ diffusivity mostly above 8 × 10^−10^ cm^2^ s^−1^, with an average value of 6.54 × 10^−10^ cm^2^ s^−1^, together with a low overpotential, indicating favorable Li⁺ diffusion kinetics in Ta_2_O_5_·*a*-LTOC-based Ni90 ASSBs (Fig. S24). Consistently, the Ni90 ASSB with Ta_2_O_5_·*a*-LTOC exhibits high-rate capacities of 192.2, 175.0, 159.8, 143.2, 130.1 and 108.6 mAh g^−1^ at 0.2, 0.5, 1, 2, 3 and 5 C, respectively (Fig. 4d). After the rate cycling test, the Ni90 ASSB continued cycling at 2 C, demonstrating long-term cycling stability with 85.3% capacity retention over 5000 cycles (Fig. 4e).

We further conducted cycling tests under various conditions to evaluate the practical applicability of Ta_2_O_5_·*a*-LTOC. Under a fast-charging/slow-discharging condition, the Ni90 ASSB with Ta_2_O_5_·*a*-LTOC exhibits stable cycling over 3500 cycles (Fig. S25). At high cathode loading, the Ni90 ASSB with Ta_2_O_5_·*a*-LTOC (21.6 mg cm^−2^) exhibits a high areal capacity (>3 mAh cm^−2^) and 80.4% capacity retention over 800 cycles in a voltage range of 2.8–4.5 V versus Li^+^/Li at 30°C (Fig. 4f). If the cathode loading is further increased to 24.9 mg cm^−2^, the Ni90 ASSB still exhibits stable cycling at 3 C for 500 cycles with a capacity retention of 92.6% in a voltage range of 2.7–4.8 V versus Li⁺/Li (Fig. S26). At a high rate of 6 C, the Ni90 ASSB delivers stable cycling performance of 4000 cycles with 81.5% capacity retention (Fig. 4g). Further increasing the rate to 15 C, the Ni90 ASSB still maintains a reversible capacity of ∼100 mAh g^−1^ and delivers stable cycling for 800 cycles with a capacity retention of 86.47% in a voltage range of 2.7–4.8 V versus Li^+^/Li (Fig. S27). Moreover, low-temperature electrochemical measurements show that the Ni90 ASSB maintains stable cycling at −10°C and 1 C for 200 cycles with a capacity retention of 94.7% in a voltage range of 2.7–4.6 V versus Li^+^/Li (Fig. S28). These results demonstrate the potential of Ta_2_O_5_·*a*-LTOC for practical high-performance ASSBs, owing to its high ionic conductivity, favorable high-voltage tolerance and robust interfacial stability.

### Universality of the interfacial percolation strategy

**Figure 5.**
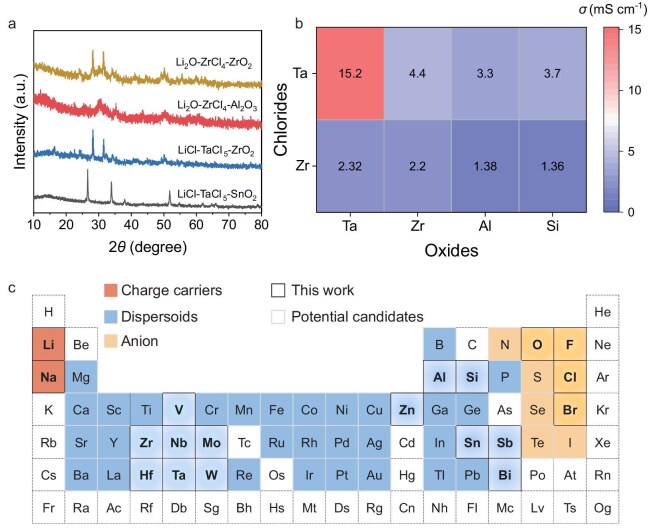
Universality of the percolative SSEs. (a) XRD patterns of typical percolative SSEs. (b) Ionic conductivities of various SSEs designed by percolation strategy. (c) Proposed potential elemental candidates for percolative SSEs.

We further validate the universality of the interfacial percolation strategy for designing SSEs. When different oxides (MO*_y_*, where M = cations such as Ta, Zr, Al and Si) are used as the dispersoid phases, all the resulting MO*_y_*-based percolative SSEs exhibit high ionic conductivities (Fig. 5a and b and Table S5). Moreover, this strategy can be extended to various metal chlorides (such as NbCl_5_ and ZrCl_4_) and alkali metal compounds (including LiCl, NaCl, LiBr and Li_2_O) as starting materials (Table S5), obtaining various percolative SSEs (MO*_y_*·LiM’OCl, where M and M' are cations) with high ionic conductivity. We display the explored and potential combinatorial elements for percolative SSEs in Fig. 5c, indicating the universality of the interfacial percolation strategy for designing novel SSEs.

## CONCLUSION

In summary, we establish an interfacial percolation design principle for inorganic SSEs, in which functional oxide dispersoids embedded in an amorphous conductive matrix enable high ionic conductivity (up to 15 mS cm^−1^ in Ta_2_O_5_·*a*-LTOC), low Li content and high-potential stability. Comprehensive experimental analyses confirm that Li-ion redistribution at the dispersoid/matrix interphases generates continuous ion percolation pathways, providing a mechanism fundamentally distinct from the Li-enrichment strategy used in conventional inorganic SSEs. Ni90 ASSBs with Ta_2_O_5_·*a*-LTOC deliver superior electrochemical performance, including high-rate capability (4000 cycles at 6 C), long cycle life (5000 cycles at 2 C) and high-loading capability (800 cycles with an areal capacity >3 mAh cm^−2^), demonstrating strong potential for practical application. The percolation strategy is demonstrated to be broadly applicable across various oxychloride SSEs, not only enabling high ionic conductivity at low Li content, but also offering tunable secondary phases for designing SSEs with desired properties (e.g. electrochemical stability), thereby enabling long-cycle, high-energy ASSBs.

## METHODS

SSEs were synthesized by high-energy ball milling with raw materials including LiCl (Alfa Aesar, 99.9%), Li_2_O (Aladin, 99.99%), TaCl_5_ (Sigma, 99.9%), Ta_2_O_5_ (Aladin, 99.99%), LiBr (Aladin, 99.99%), LiF (Aladin, 99.99%), NaCl (Aladin, 99.99%), Nb_2_O_5_ (Aladin, 99.99%) and NbCl_5_ (Aladin, 99.99%). For the Li_1.6_Ta_2.4_O_3_Cl_7.6_, stoichiometric amounts of powders of LiCl, TaCl_5_ and Ta_2_O_5_ were milled in ZrO_2_ jars (80 mL) with ZrO_2_ balls using Pulverisette 7PL (Fritsch GmbH). The starting material for each compound was weighed in an argon-filled glove box (H_2_O <0.01 ppm, O_2_ <0.1 ppm) and sealed into the ball mill jars. The milling was first conducted at 150 rpm for 1 h to mix the starting materials and then milled at a higher speed of 550 rpm for 20 h (milling for 5 mins and pause for 5 mins) to get the final powders. The MO*_y_*·LiM’OCl (where M and M' are cations) were synthesized with the same methods.

More detailed methods can be found in the online Supplementary data.

## Supplementary Material

nwag411_Supplemental_File
