# Supplementary material for: Interfacial percolation enables high-conductivity lean-lithium oxychloride electrolytes for all-solid-state batteries
Source: Natl Sci Rev. 2026 Jul 3;13(15):nwag411. doi: 10.1093/nsr/nwag411 (PMC13455620; doi:10.1093/nsr/nwag411)
Supplement: Supplementary file 1 [file nwag411_supplemental_file.pdf]

Supplementary Information

Interfacial Percolation Enables High-Conductivity Lean-Lithium Oxychloride Electrolytes for All-Solid-State Batteries

Deyuan Li[1], Guangwen Zhang[1], Boyuan Xu[2], Feifei Luo[1], Kongying Zhu[3], Guohong Liang[1], Tianran Yan[4], Weiqi Hou[1], Liang Zhang[4], Qisheng Wu[2], Chunpeng Yang[1]*

[1]Tianjin Key Laboratory of Advanced Carbon and Electrochemical Energy Storage, School of Chemical Engineering and Technology, Tianjin University, Tianjin 300350, China

[2]Division of Energy & Environmental Materials, Suzhou Laboratory, Suzhou, Jiangsu 215123, China

[3]Analysis and Test Center, Tianjin University, Tianjin 300350, China

[4]Institute of Functional Nano & Soft Materials, Jiangsu Key Laboratory of Advanced Negative Carbon Technologies, Soochow University, Suzhou 215123, China

*E-mail: cpyang@tju.edu.cn

This file includes:

Materials and methods

Figures S1–S28

Tables S1–S5

Supplemental References

Materials and methods

Materials characterizations

Powder XRD measurements were performed using a Rigaku SmartLab diffractometer with Cu-Kα radiation (λ = 1.5406 Å) while Kapton film was used to prevent air exposure. Synchrotron-based micro X-ray powder diffraction (SXRD) measurement was conducted at the ID13 beamline at the European Synchrotron Radiation Facility (ESRF) in Grenoble. 2D area detector Eiger 4M was used to collect diffraction patterns covering reciprocal space q ranging from 0.08 to 6.4 $Å^{-1}$ with a photon energy of 13 keV (λ = 0.953 Å).

Raman spectra were measured using the Raman spectrometer system (Horiba, 532 nm laser), while the electrolyte powders were tested inside a homemade 2032 cell case with quartz glass sealed. Fourier-transform infrared (FT-IR) spectra were collected using a Thermo Scientific Nicolet iS50 spectrometer.

The sample was loaded using a TEM cryogenic holder (Gatan, Elsa™ 698), where TEM grids are immersed in liquid nitrogen and then subsequently mounted onto the holder via a cryo-transfer workstation (−170 °C). Microscopic morphology, elemental analysis, and high-resolution TEM were performed by the transmission electron microscopy (TEM, JEM-F200) equipped with an EDS system (JEOL). Atomic-resolution HAADF-STEM images were collected by spherical aberration-corrected TEM (JEM-ARM200F) with an acceleration voltage of 200 kV. SR-EELS was conducted using a 300 kV TEM (JEM-ARM300F2 FHP) equipped with an EELS system in STEM mode.

The XAS analyses were performed at the hard X-ray beamline station in the Shanghai Synchrotron Radiation Facility (SSRF, 31124.02.SSRF.BL11B). The XAS data were analyzed with the Athena software, then the EXAFS data were processed using the Artemis program[1].

The solid-state ^{6}Li MAS-NMR spectra analyses were recorded on JEOL JNM ECZ600R at a spinning speed of 15 kHz. All the samples were sealed in ZrO_2 tubes in an Ar-filled glovebox (H_2O and O_2 < 0.1 ppm). The ^{6}Li chemical shift was calibrated with solid LiCl powder at 0 ppm. The ^{6}Li exchange analysis was conducted on a $^{6}LiIn$ | LPSC | $Ta_2O_5 \cdot a$-LTOC | LPSC |

^{6}LiIn symmetric battery, while the LPSC was utilized to prevent the side reaction between $Ta_2O_5 \cdot a$-LTOC and LiIn anode. The batteries were cycled for 16 h charging (1st cycle), or 16 h charge → 16 h discharge→ 16 h charge (2nd cycle) at a constant current of 0.1 mA to ensure the ^{6}Li atoms in the anode are exchanged with the Li atoms in $Ta_2O_5 \cdot a$-LTOC. After testing, the $Ta_2O_5 \cdot a$-LTOC in the middle layer was selected for testing. Room-temperature ^{7}Li NMR T_1 and T_2 relaxation measurements were conducted on JEOL JNM ECZ600R. T_1 was determined using a saturation-recovery pulse sequence, while T_2 was determined using a spin-echo pulse sequence. The relaxation times were obtained by fitting the corresponding recovery and decay profiles with exponential functions.

The XPS analyses were conducted by ThermoFisher Scientific (K-Alpha+, ESCALAB Xi+), and the binding energies were calibrated by setting the measured binding energy of C 1s to 284.8 eV. All the samples were prepared using a glovebox-compatible inert gas sample transfer system in an Ar-filled glovebox (H_2O and O_2 < 0.1 ppm), ensuring complete isolation from air. The Li concentration was determined based on the density of a pressed pellet.

Electrochemical characterizations

The ionic conductivity of the prepared electrolyte was evaluated by EIS with an amplitude of 10 mV and a frequency range from 1 Hz to 7 MHz using the SP-300 (Bio-Logic). The electrolyte powders (~130 mg) were cold-placed between two stainless rods and pressed into a 10 mm diameter pellet under ~380 MPa.

ASSB assembly and test: First, ~60 mg sulfide SSEs (LPSC, canrd) were pressed at ~200 MPa in a PEEK cylinder (10 mm diameter), and then ~50 mg of the prepared SSE powders were spread on the sulfide SSEs and pressed for ~300 MPa. The commercial uncoated single-crystal Ni90 (BASF Shanshan Battery Materials Co., LTD), oxychloride SSEs, and vapor-grown carbon fiber (canrd) were weighed in a weight ratio of 70: 30: 3 and then mixed uniformly using the vortex oscillator for 10 min to prepare the cathode composites. Cathode composite was spread evenly on the side of oxychloride SSEs and then pressed for ~380 MPa. Subsequently, a piece of In foil (10 mm diameter, 100 μm thickness) was placed on the side of the LPSC electrolyte layer, followed by attaching a piece of thin Li foil (8 mm diameter, 100 μm thickness)

and pressing under 60 MPa. All the cell preparation processes were conducted in the Ar-filled glovebox. The cells were cycled at 30 °C or RT using the LAND battery testing system. The rate current density was calculated based on conventional 1 C value of 200 mA g^{-1} for Ni90.

The linear sweep voltammetry (LSV) electrochemical stability window analyses were conducted on LiIn | LPSC | SSE | SSE+VGCF batteries with a scan rate of 0.1 mV s^{-1} using SP-300 (Bio-logic) at room temperature. The ratio of SSE and VGCF was 9: 1, ~100 mg LPSC, 50 mg SSE, and 10 mg SSE+VGCF mixture were used for LiIn | LPSC | SSE | SSE+VGCF cells. The electrochemical floating test was conducted on the Ni90 ASSB with $Ta_2O_5 \cdot a$-LTOC, which was first charged to 4.3 V vs. Li^+/Li, followed by stepwise voltage holds from 4.3 to 4.8 V vs. Li^+/Li in 0.1 V increments, each held for 20 h. The diffusion coefficient (D_σ) was calculated using the Nernst–Einstein relation ($D_\sigma = \frac{\sigma k_B T}{|nq^2|}$).

Supplemental Figures

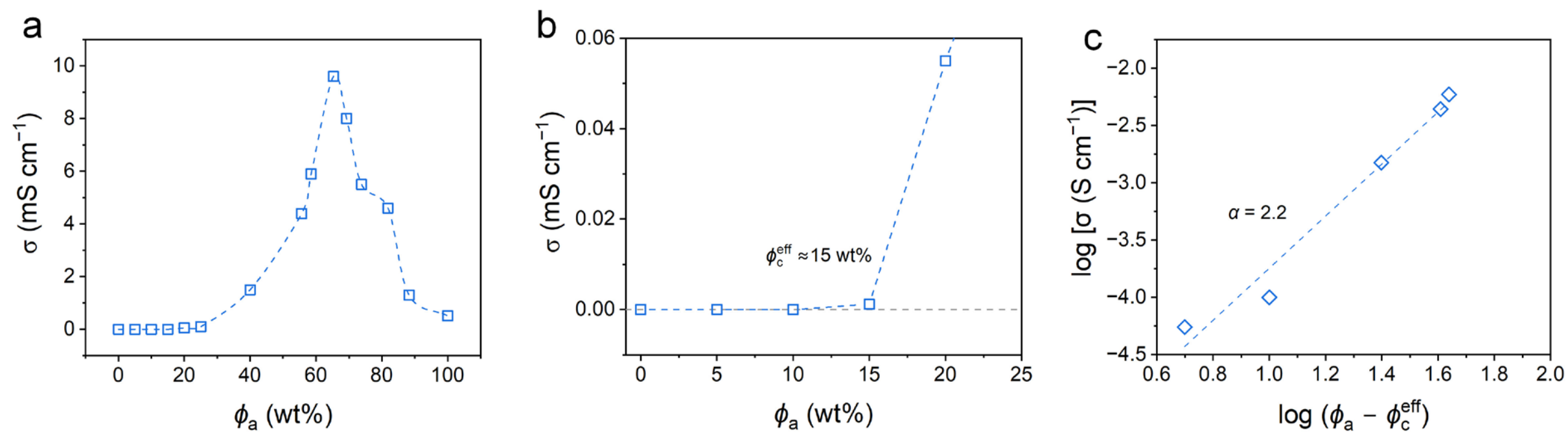

Figure S1. (**a**) Ionic conductivities of SSEs prepared from 1.63 LiCl: 1.2 $TaCl_5$: *k* Ta_2O_5 as a function of estimated amorphous phase fraction. (**b**) Conductivity near the effective percolation threshold, showing $\phi_c^{eff} \approx 15$ wt%. (c) Log–log fitting based on $\sigma = A(\phi_a - \phi_c^{eff})^{\alpha}$, giving an apparent exponent of $\alpha \approx 2.2$.

We analyzed the ionic conductivity as a function of the amorphous phase fraction (ϕ_a). According to the percolation-type scaling relationship, the conductivity above the threshold follows the equation:[2, 3]

$$\sigma = A(\phi_a - \phi_c^{eff})^{\alpha} \tag{1}$$

where σ is the ionic conductivity, A is a scaling pre-factor, ϕ_a is the amorphous phase mass fraction, ϕ_c^{eff} is the effective percolation threshold, and α is the apparent percolation exponent.[2, 3] In this analysis, ϕ_a was estimated from the precursor mass fraction and used as an effective composition descriptor for the amorphous/interfacial conductive regions, assuming comparable Ta_2O_5 participation in the interfacial reaction among different compositions. The log–log fitting of σ versus $\phi_a - \phi_c^{eff}$ yields an apparent percolation exponent of $\alpha \approx 2.2$ (Figure S1c), supporting a power-law transition from isolated amorphous/interfacial regions to connected Li^+ transport pathways.

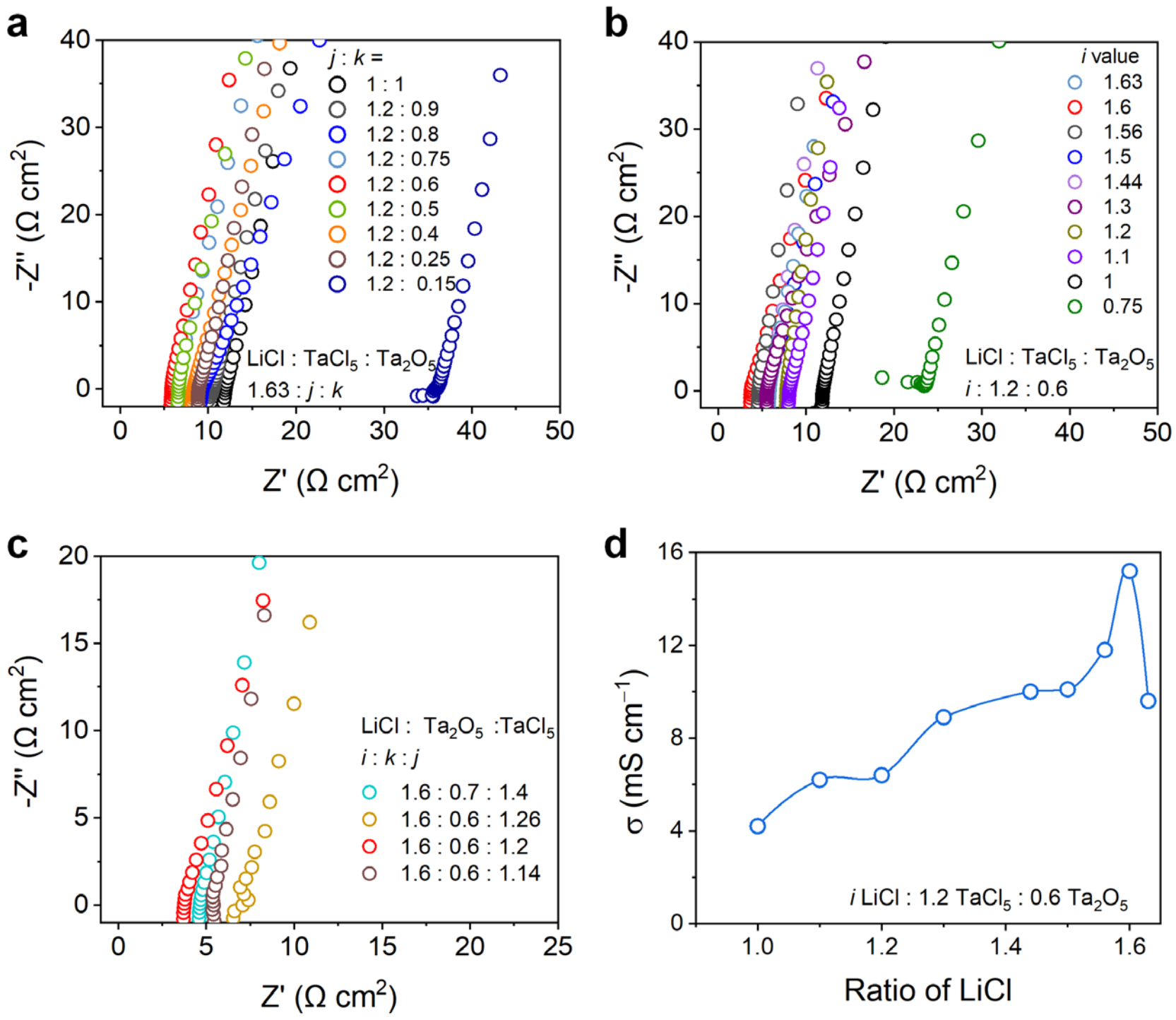

Figure S2. Composition regulation of $Li_iTa_{j+2k}O_{5k}Cl_{i+5j}$ SSEs. (a–c) Nyquist plots of SSEs with varied precursor ratios: (a) 1.63LiCl: jTaCl$_5$: kTa$_2$O$_5$, (b) iLiCl: 1.2TaCl$_5$: 0.6Ta$_2$O$_5$, and (c) 1.6LiCl: jTaCl$_5$: kTa$_2$O$_5$. (d) Ionic conductivities of iLiCl: 1.2TaCl$_5$: 0.6Ta$_2$O$_5$ SSEs.

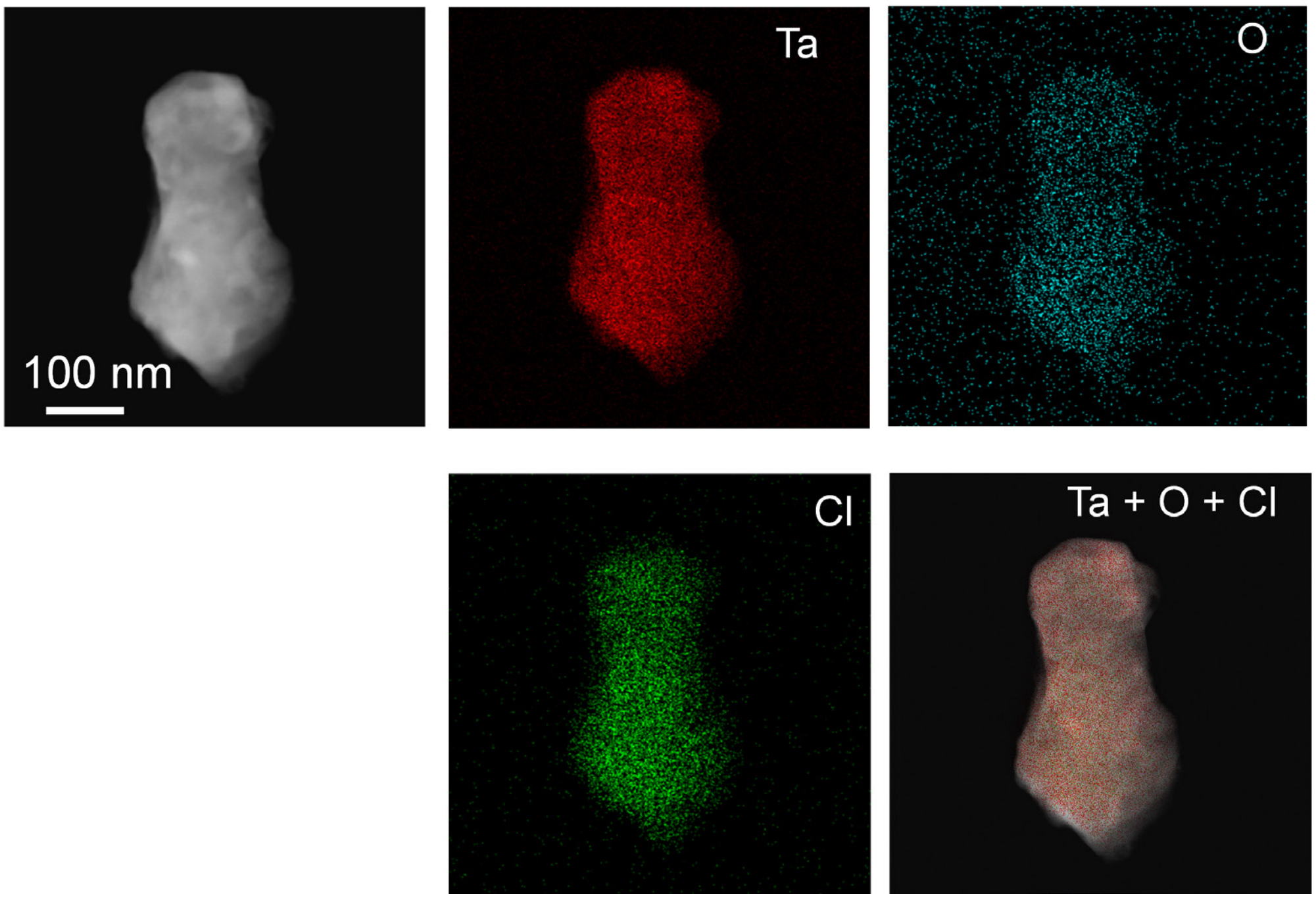

Figure S3. EDS mapping images of $Ta_2O_5 \cdot a$-LTOC.

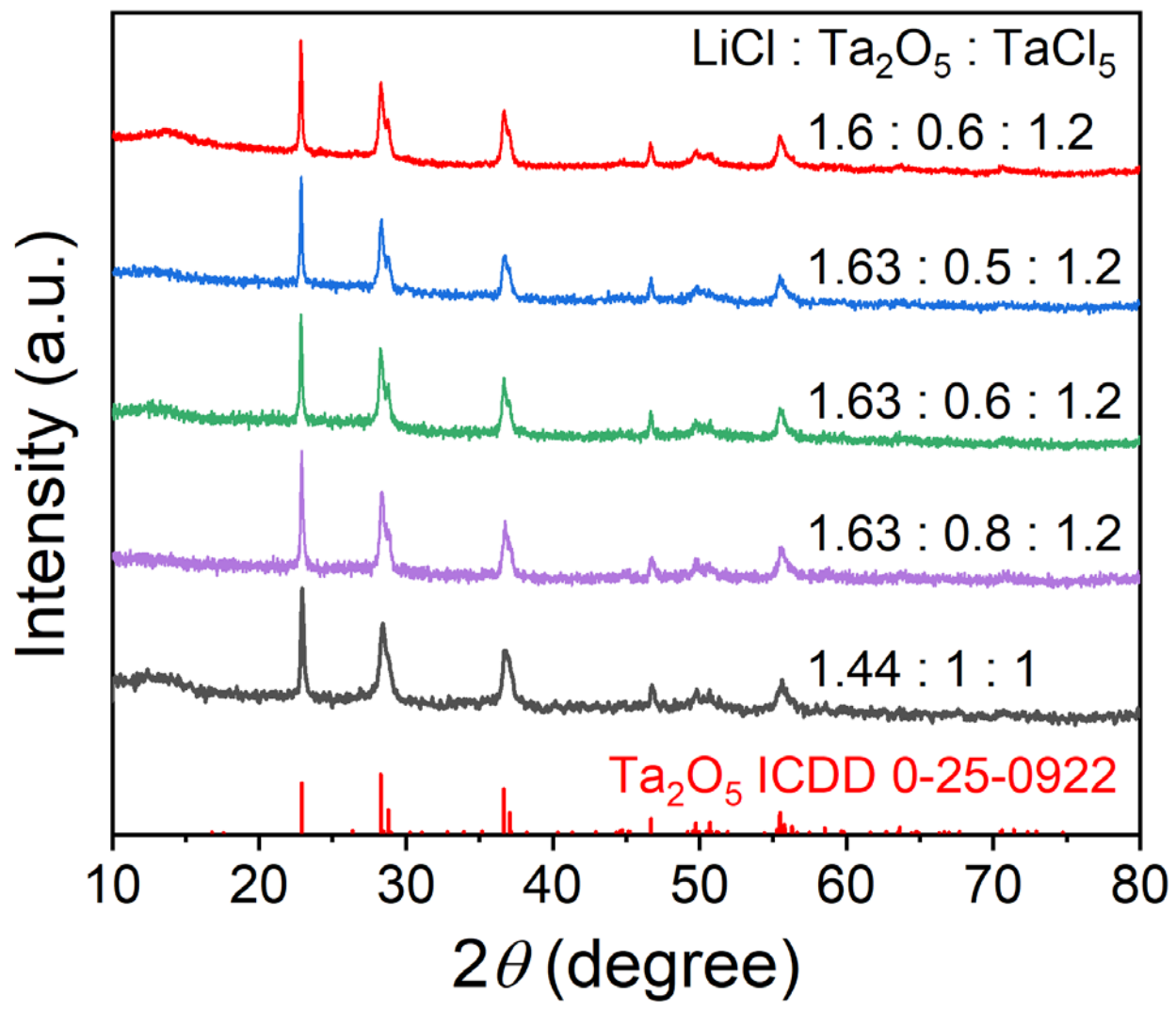

Figure S4. Typical XRD patterns of *i* LiCl: *j* $TaCl_5$: *k* Ta_2O_5.

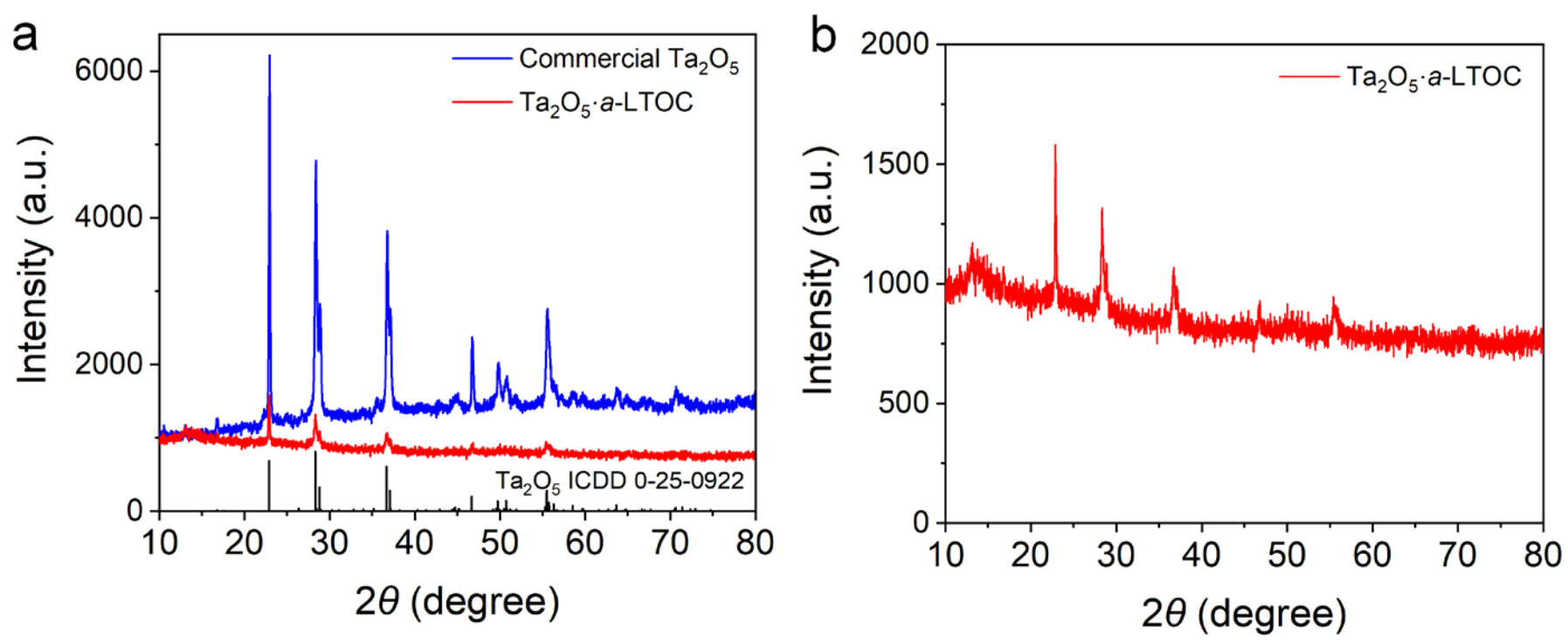

Figure S5. XRD patterns of $Ta_2O_5{\cdot}a$-LTOC and commercial Ta_2O_5. (a) Same-intensity-scale comparison of $Ta_2O_5{\cdot}a$-LTOC and commercial Ta_2O_5. (b) Separately plotted XRD pattern of $Ta_2O_5{\cdot}a$-LTOC showing broad diffuse scattering and residual Ta_2O_5 reflections. All patterns were collected from 10° to 80° at 3° min^{-1}.

Compared with commercial Ta_2O_5, $Ta_2O_5{\cdot}a$-LTOC shows much weaker and relatively broadened Ta_2O_5-related reflections under identical laboratory XRD conditions, suggesting reduced crystalline Ta_2O_5 contribution and possible structural disorder/crystallite-size reduction after ball milling/reaction. The broad diffuse scattering of $Ta_2O_5{\cdot}a$-LTOC further supports the presence of an amorphous phase.

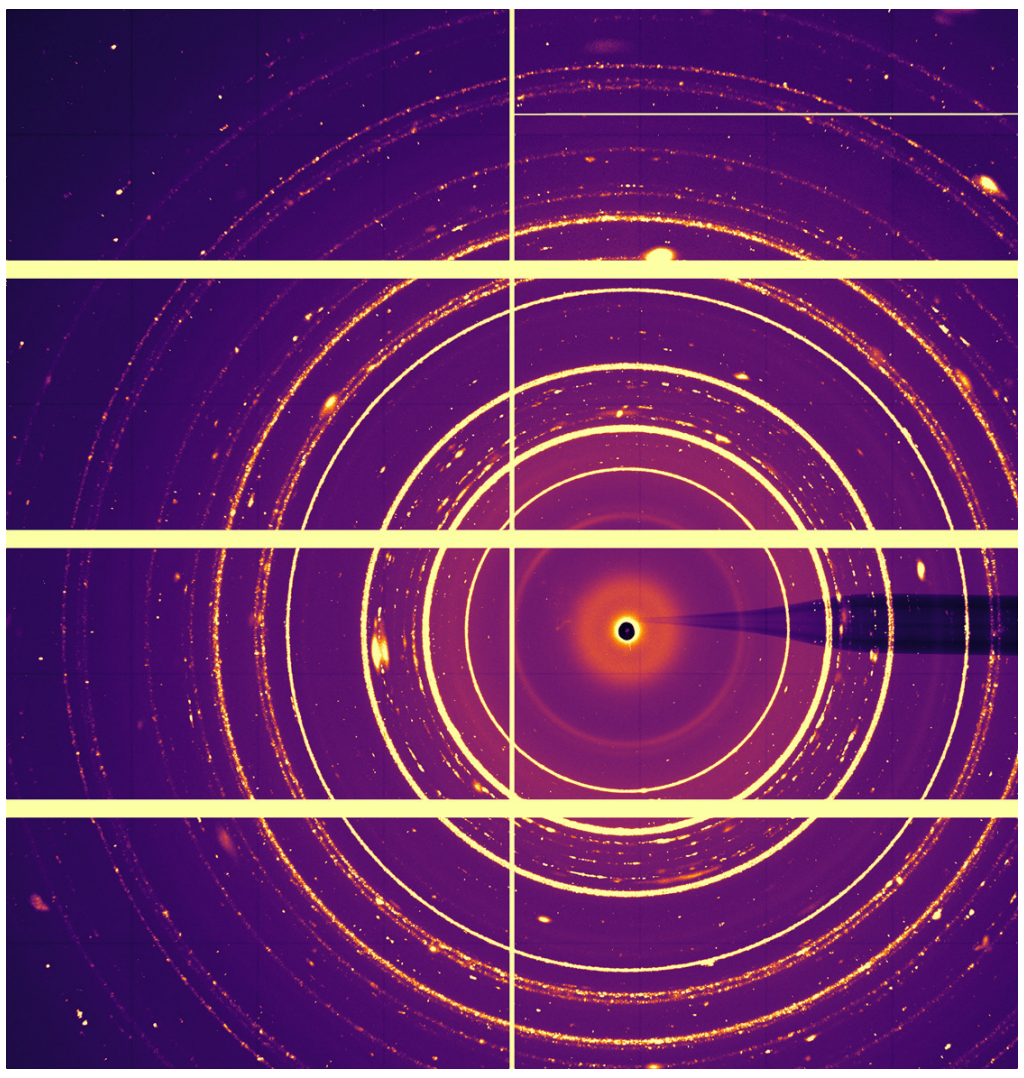

Figure S6. 2D SXRD pattern of $Ta_2O_5 \cdot a$-LTOC.

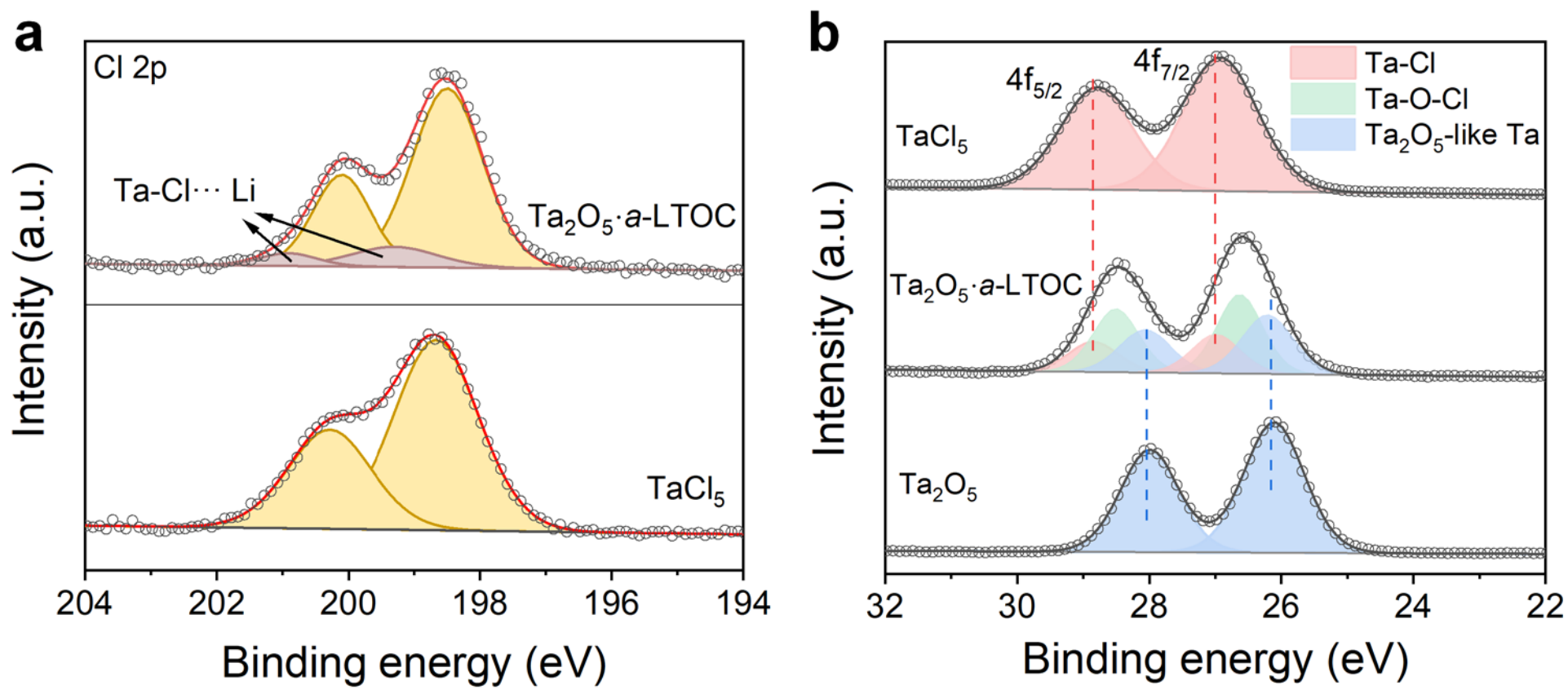

Figure S7. (**a**) Cl 2p, and (**b**) Ta 4f XPS spectra of the samples.

Cl atoms, due to their larger ionic radius, higher ionic polarizability, and weak binding energy with Li ions, mainly originate from unsaturated Ta-Cl···Li bonds and act as terminal ligands, thereby facilitating ion migration.[4-6] The fitted Ta 4f spectrum of $Ta_2O_5 \cdot a$-LTOC reveals the coexistence of Ta_2O_5-like Ta, Ta–Cl-related coordination, and mixed Ta–O–Cl coordination.[7] The mixed Ta–O–Cl component is located between the $TaCl_5$ and Ta_2O_5 reference components, consistent with an intermediate Ta local environment involving both O and Cl coordination. These results support multiple Ta local environments in $Ta_2O_5 \cdot a$-LTOC.

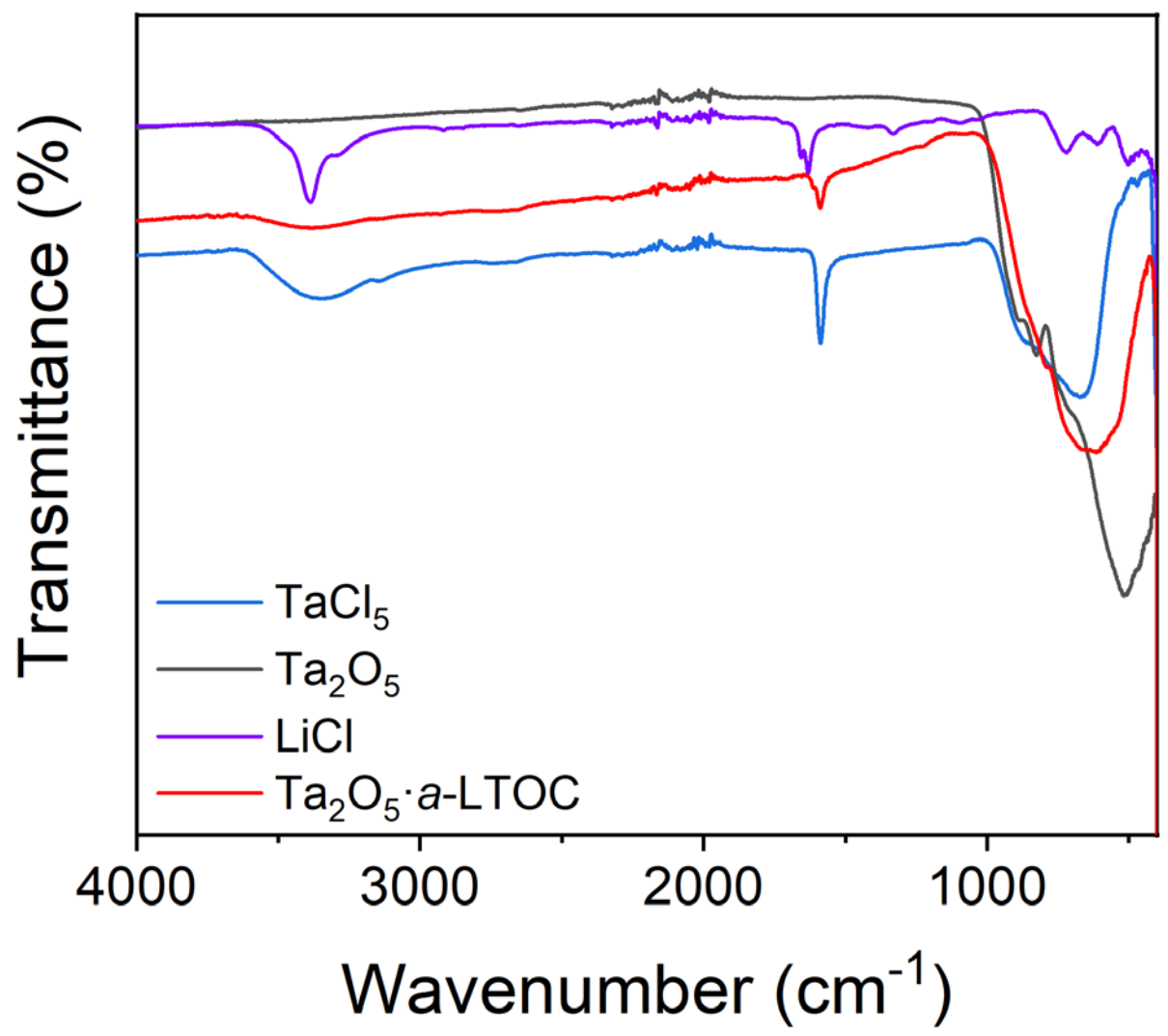

Figure S8. FTIR spectra of the samples.

Fourier transform infrared (FTIR) spectrum of Ta_2O_5·*a*-LTOC further reveals that the Ta-Cl vibrational frequency shifts from 665 to 630 cm^{-1} with peak broadening, indicating a change in Ta-Cl bond environment likely associated with O atoms insertion, amorphization, or the presence of multiple coordination environments.[8] Additionally, the peak at 829 cm^{-1} of Ta_2O_5 decreases in intensity and red shifts to 792 cm^{-1} in Ta_2O_5·*a*-LTOC, indicating a change in the internal Ta-O-Ta vibrational mode, possibly due to the formation of $[TaCl_{5-x}O_x]^{x-}$ (x<5) coordination.[8, 9]

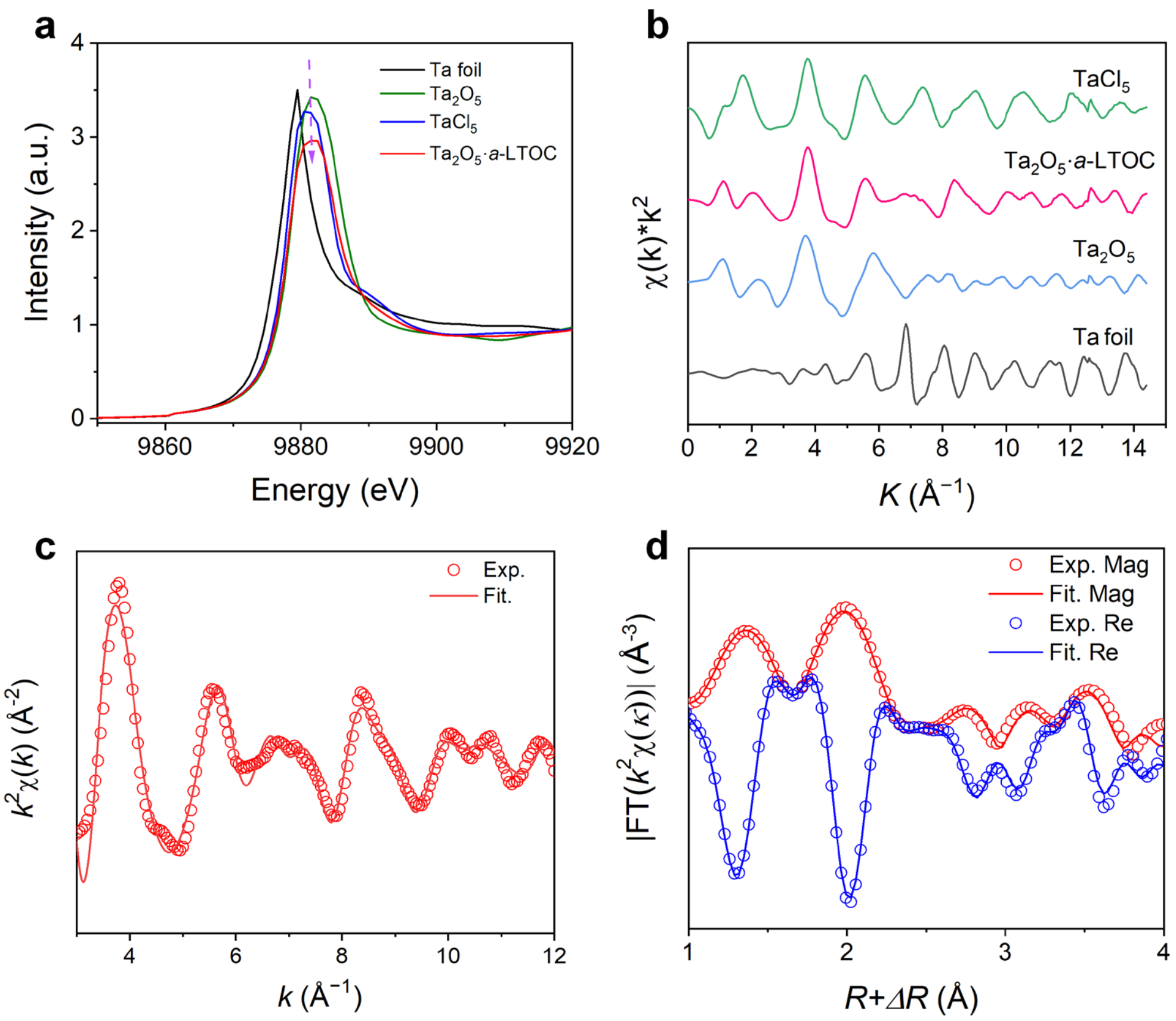

Figure S9. (**a**) XANES, (**b**) EXAFS for $Ta_2O_5 \cdot$*a*-LTOC, $TaCl_5$, Ta_2O_5, and Ta foil at Ta L_3-edge with k^2 weighting. (**c**) Fitted k-space, and (**d**) R-space curves in Ta L_3-edge for $Ta_2O_5 \cdot$*a*-LTOC.

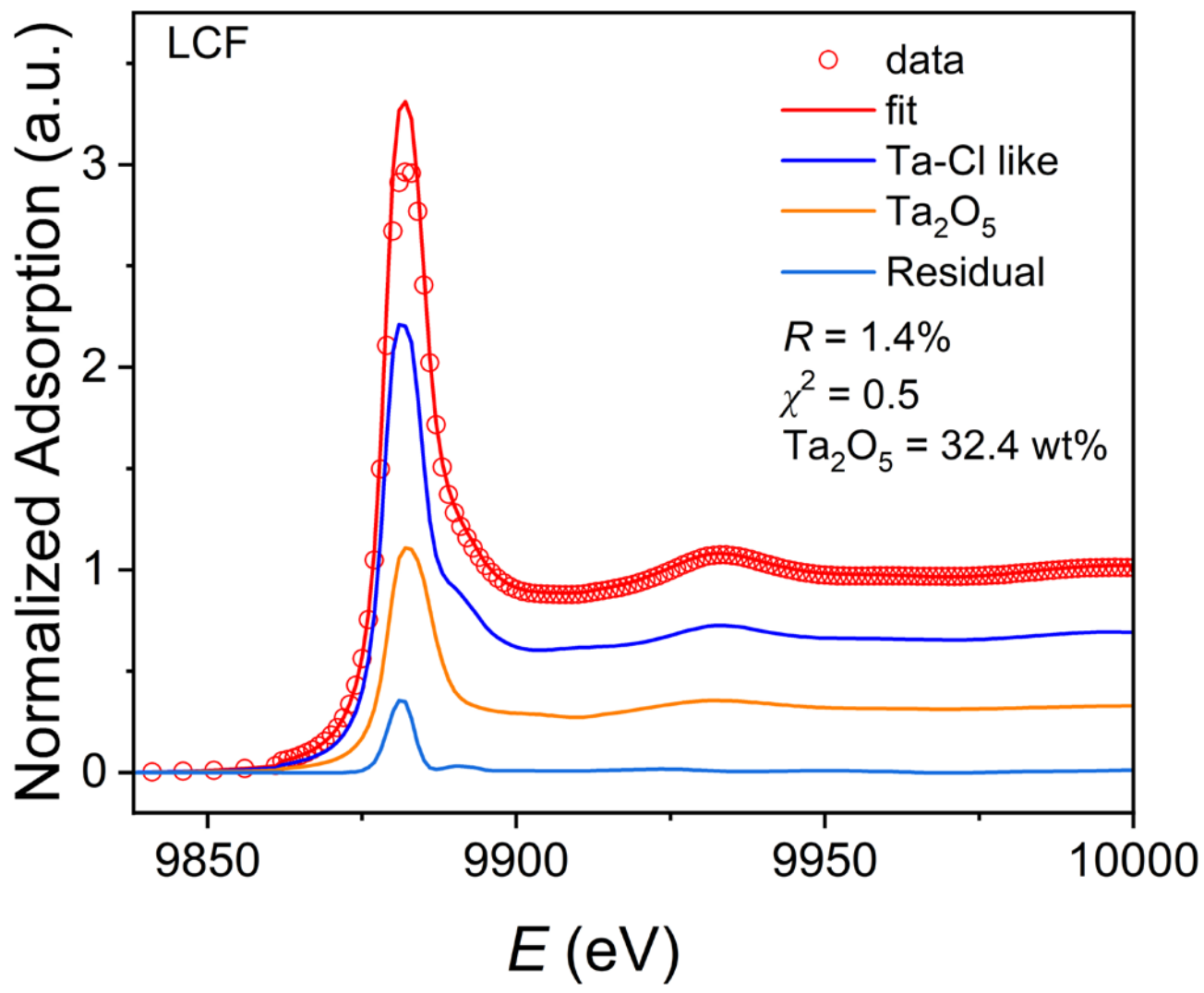

Figure S10. Ta L_3-edge XANES linear combination fitting (LCF) of the $Ta_2O_5 \cdot a$-LTOC sample, revealing that the Ta_2O_5 fraction in the $Ta_2O_5 \cdot a$-LTOC sample is 32.4 wt%.

Ta L_3-edge XANES linear combination fitting (LCF) was performed to estimate the Ta_2O_5-like contribution in $Ta_2O_5 \cdot a$-LTOC and clarify the origin of the Ta-O signals. The fitting gives an estimated Ta_2O_5-like contribution of 32.4 wt%, slightly lower than the nominal Ta_2O_5 fraction in the starting composition (34.76 wt%). This result suggests that most Ta_2O_5 remains as residual Ta_2O_5-like oxide/dispersoid environments, while a small fraction of Ta_2O_5 participates in the reaction and contributes to amorphous/interfacial oxychloride environments. Accordingly, the Ta-O signals in XAS should be attributed to both Ta-O bonds in residual Ta_2O_5 nanocrystals and mixed Ta-O-Cl local environments within the amorphous matrix/interphase. This analysis avoids assigning the Ta-O contribution solely to either the dispersed Ta_2O_5 phase or the amorphous matrix, and supports the role of Ta_2O_5 as a functionalized oxide dispersoid involved in interphase formation.

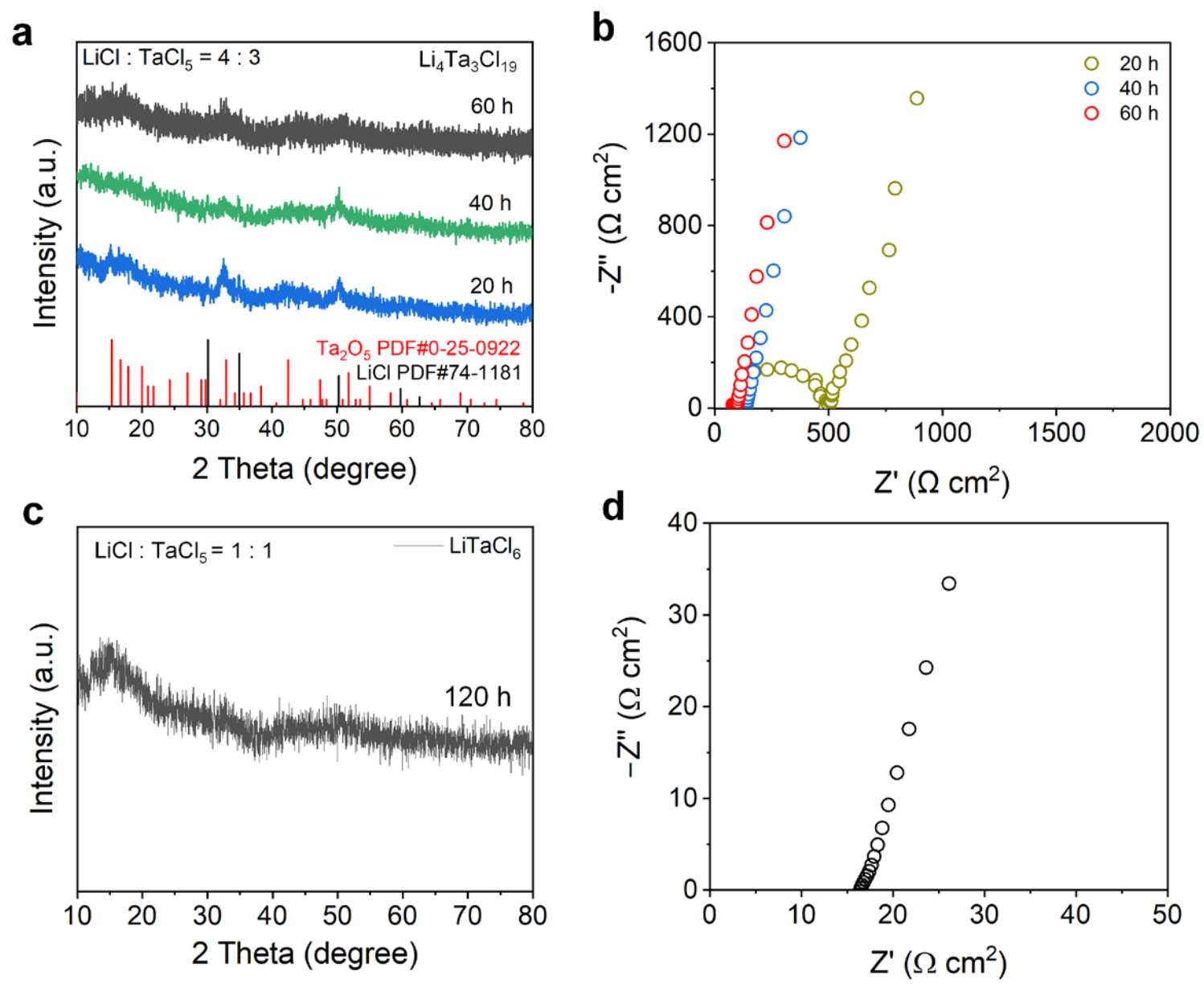

Figure S11. (**a**) XRD patterns and corresponding (**b**) EIS plots of $Li_4Ta_3Cl_{19}$ after ball milling for different times. (**c**) XRD pattern and corresponding (**d**) EIS plot of $LiTaCl_6$ after ball milling for 120 h.

When $TaCl_5$ and LiCl are directly subjected to ball milling to form $Li_4Ta_3Cl_{19}$ without the addition of Ta_2O_5, the reaction remains incomplete even after extended milling, as evidenced by residual $TaCl_5$/LiCl impurities and low ionic conductivities. The Ta_2O_5-free $Li_4Ta_3Cl_{19}$ shows a much lower ionic conductivity of only 0.51 mS cm^{-1}, far below that of Ta_2O_5·*a*-LTOC (15.2 mS cm^{-1}), indicating that the high conductivity of Ta_2O_5·*a*-LTOC cannot be attributed to the Ta_2O_5-free Li-Ta-Cl matrix alone. Similarly, $LiTaCl_6$ obtained after 120 h of ball milling still shows limited ionic conductivity compared with Ta_2O_5·*a*-LTOC. In contrast, the introduction of Ta_2O_5 significantly accelerates the reaction and enhances the ionic conductivity. This improvement may be related to the reactive oxide/halide interfaces provided by Ta_2O_5 during high-energy ball milling. These interfaces can promote local mechanochemical activation and reconstruction of Ta-O/Ta-Cl coordination environments, facilitating the formation of mixed Ta-O-Cl units and amorphous oxychloride regions. With continued milling, these local interfacial regions may progressively connect into a more continuous amorphous Ta-O-Cl network, thereby accelerating phase formation and providing more effective Li^+-transport pathways.[10-12]

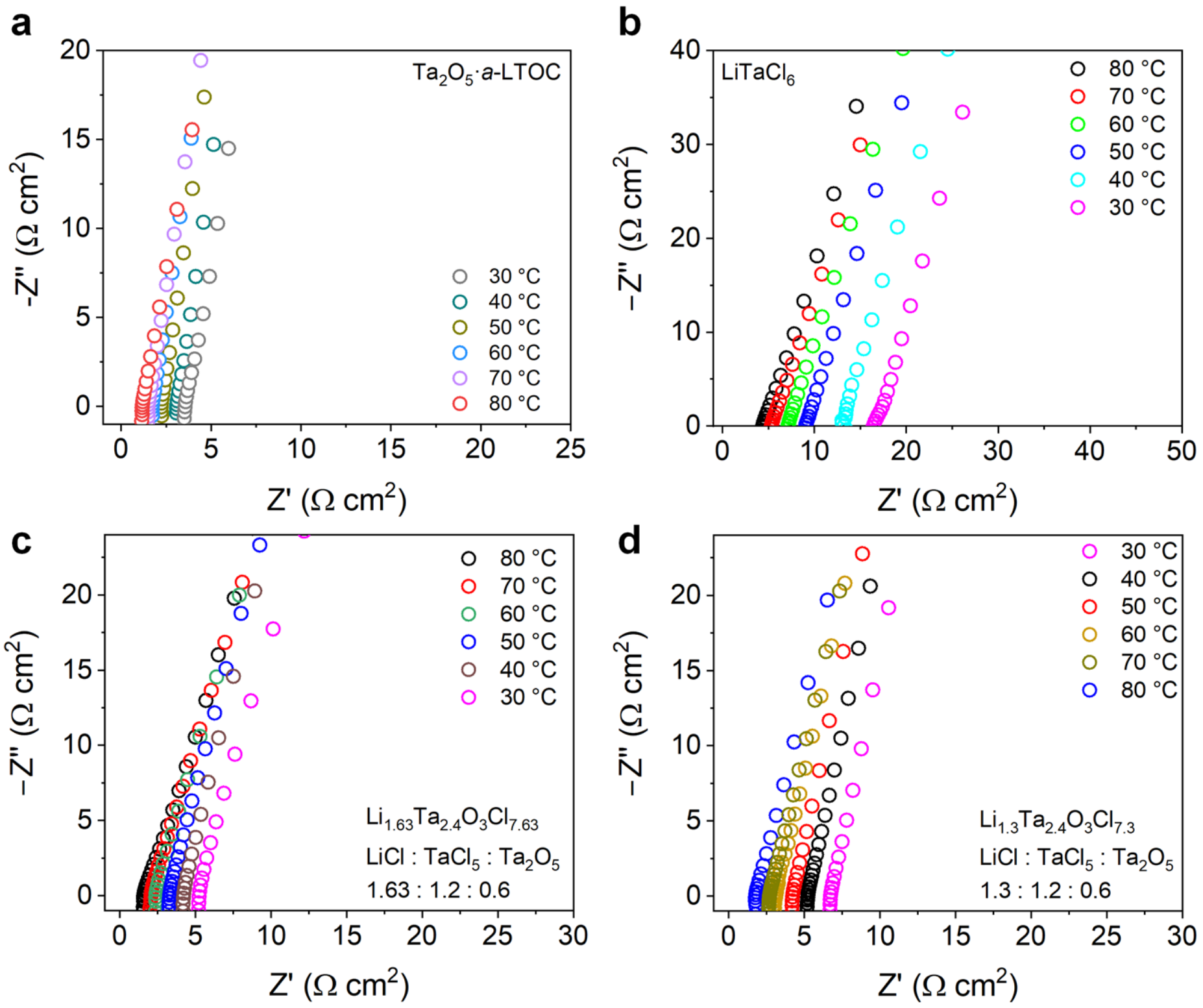

Figure S12. Temperature-dependent EIS Nyquist plots of (**a**) Ta_2O_5·*a*-LTOC, (**b**) $LiTaCl_6$, (**c**) $Li_{1.63}Ta_{2.4}O_3Cl_{7.63}$, and (**d**) $Li_{1.3}Ta_{2.4}O_3Cl_{7.3}$.

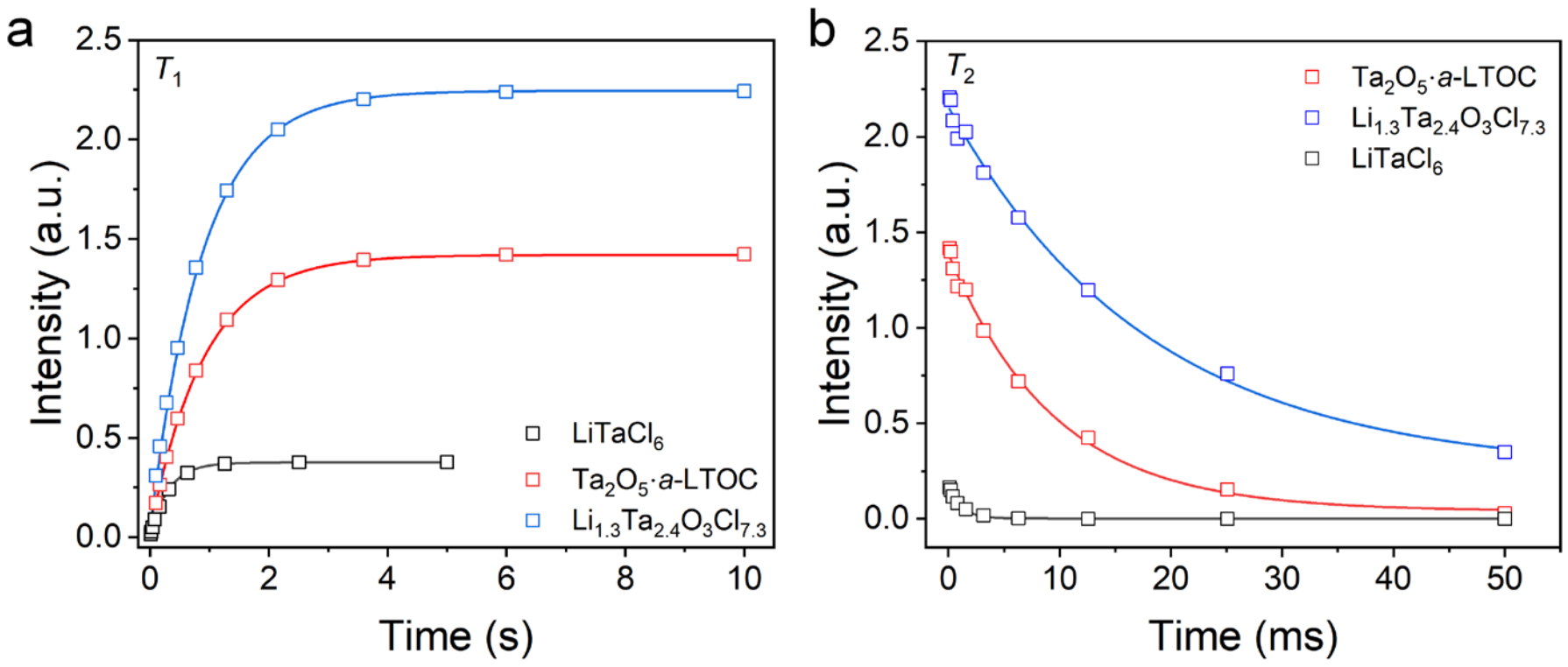

Figure S13. Room-temperature ^{7}Li relaxation curves of representative SSEs: (**a**) T_1 recovery curves and (**b**) T_2 decay curves for Ta_2O_5·*a*-LTOC, $Li_{1.3}Ta_{2.4}O_3Cl_{7.3}$, and $LiTaCl_6$.

To further probe composition-dependent local Li relaxation characteristics, we performed room-temperature ^{7}Li NMR T_1 and T_2 relaxation measurements for representative compositions (Figure S13 and Table S3). T_1 and T_2 are sensitive to local Li relaxation processes and structural heterogeneity, and therefore provide supplementary local dynamic descriptors rather than direct predictors of macroscopic conductivity.[13, 14] Ta_2O_5·*a*-LTOC shows a longer T_1 than $LiTaCl_6$ and a relatively long T_2, suggesting distinct local Li relaxation characteristics in the amorphous/interfacial environment. However, $Li_{1.3}Ta_{2.4}O_3Cl_{7.3}$ exhibits the longest T_2, while its conductivity remains lower than that of Ta_2O_5·*a*-LTOC. This indicates that local Li relaxation characteristics alone do not determine macroscopic conductivity. Therefore, the higher conductivity of Ta_2O_5·*a*-LTOC should be understood as the combined result of lower E_a, prefactor-related transport contribution, higher conductivity-to-Li-content ratio, distinct local Li relaxation characteristics, and continuous interfacial percolation pathways.

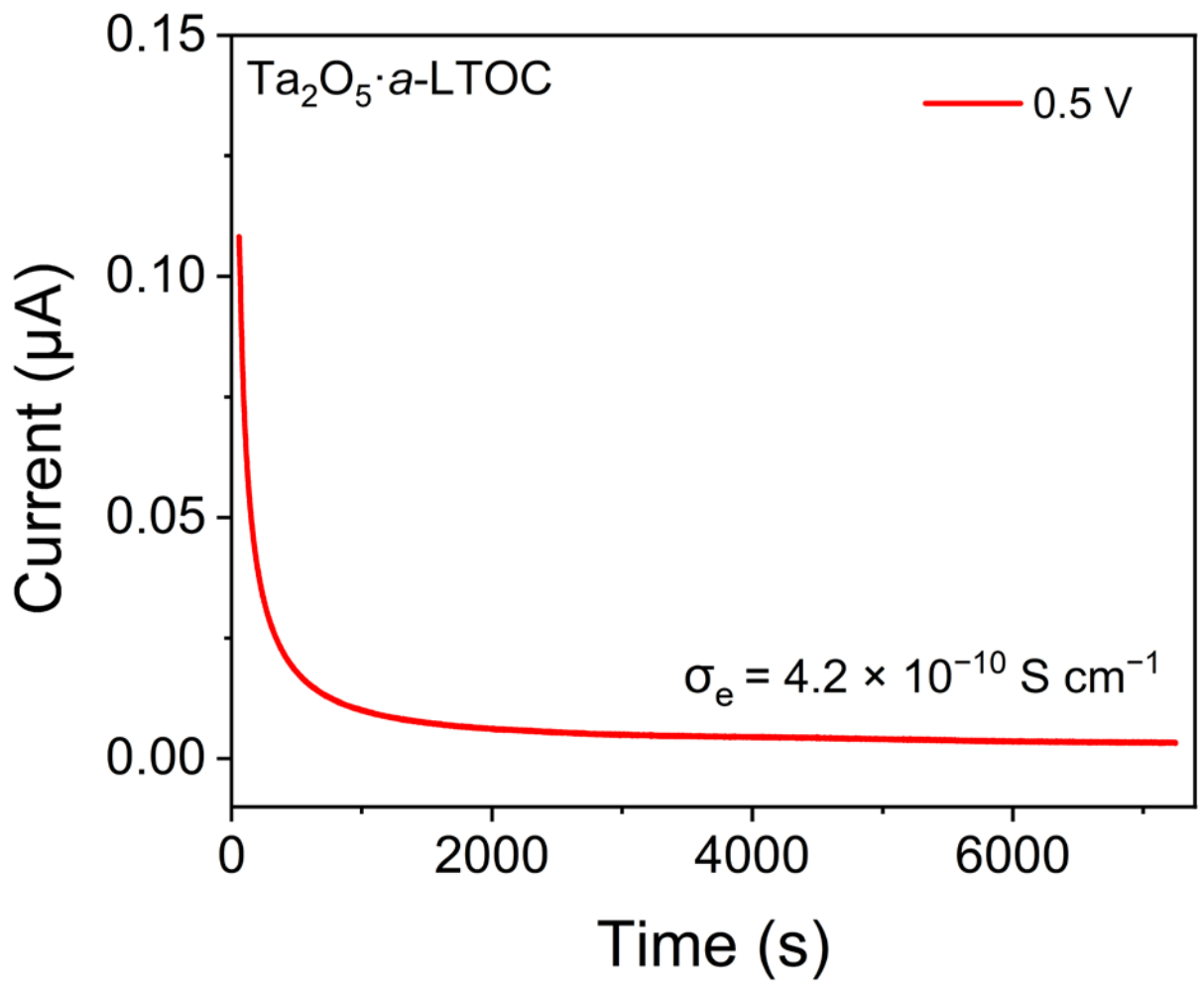

Figure S14. DC polarization curve of Ta_2O_5·*a*-LTOC for the electronic conductivity measurement.

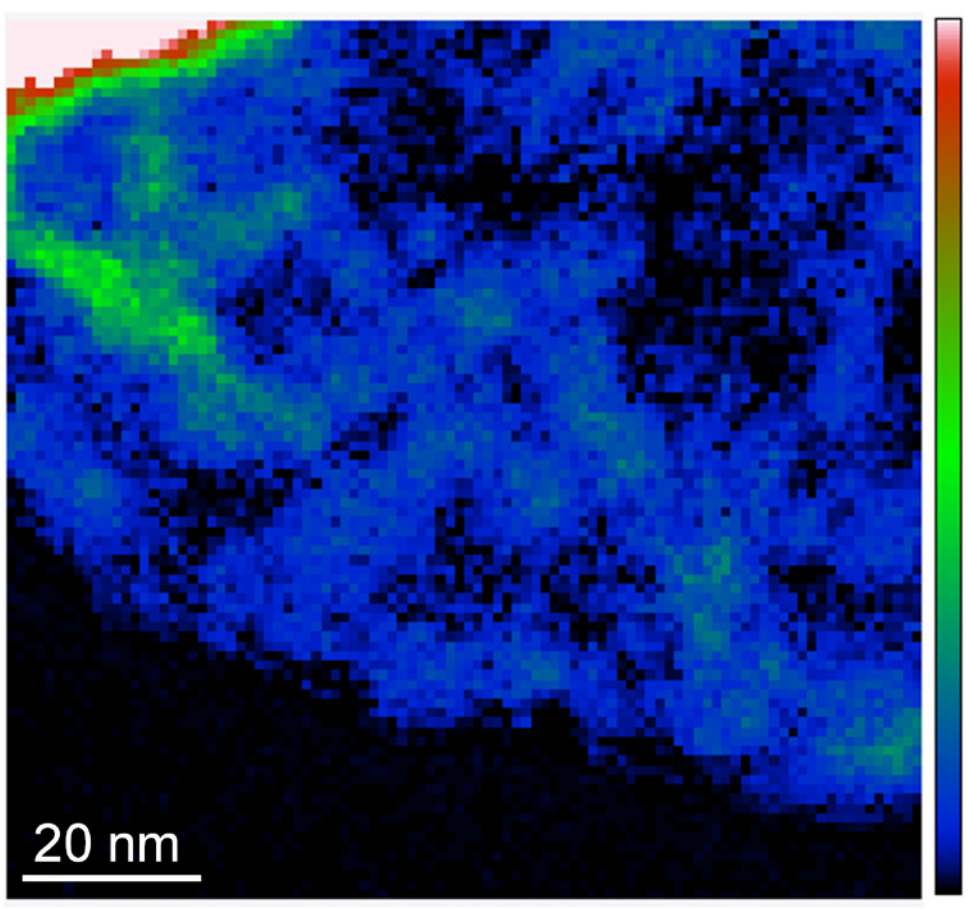

Figure S15. SR-EELS mapping of O acquired from Fig. 3d.

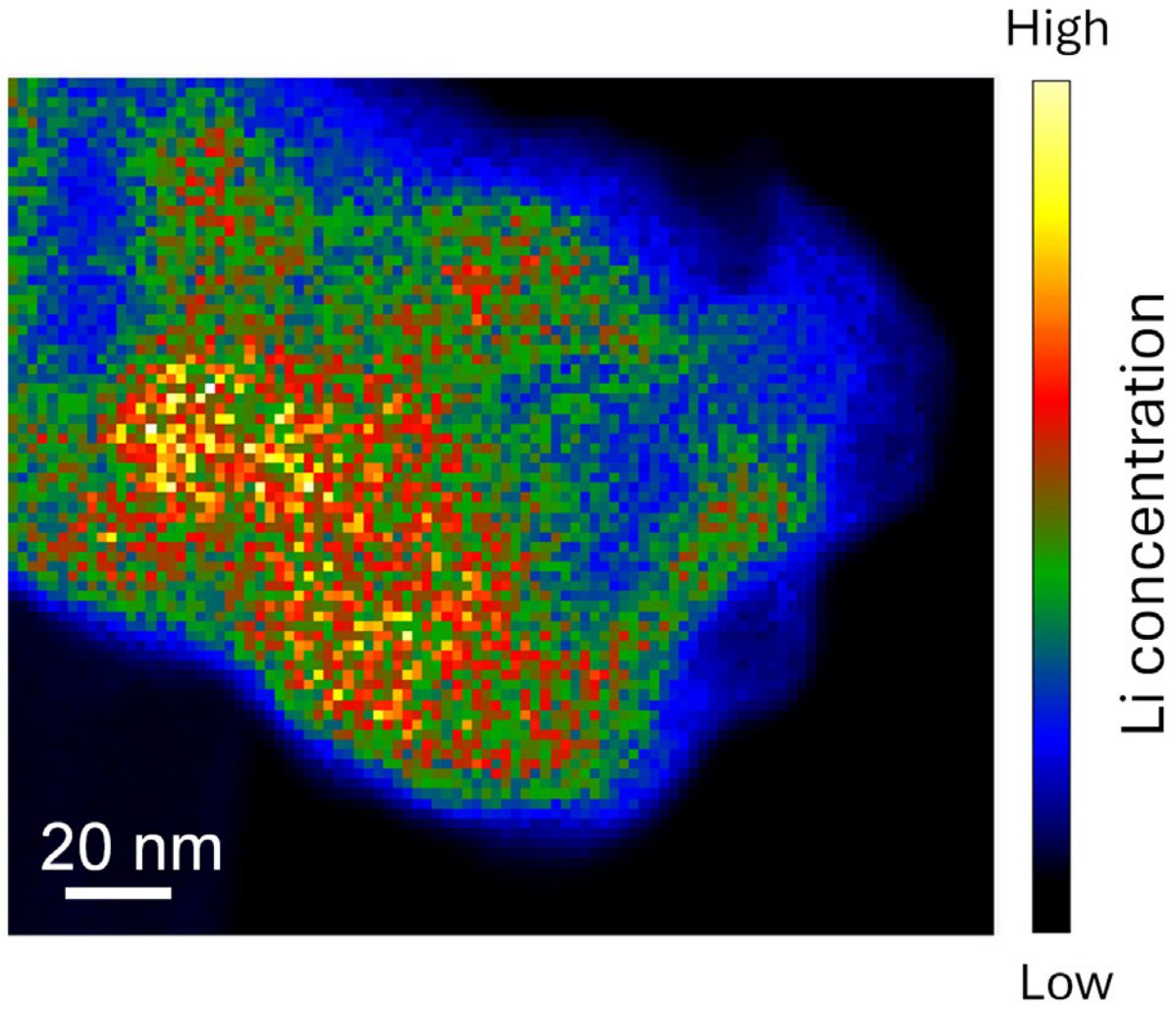

Figure S16. Li SR-EELS mapping results of low-conductivity $Li_{1.63}Ta_{1.5}O_{0.75}Cl_{7.63}$ with low Ta_2O_5 content (1.63LiCl: 1.2$TaCl_5$: 0.15Ta_2O_5, 1.3 mS cm^{-1}).

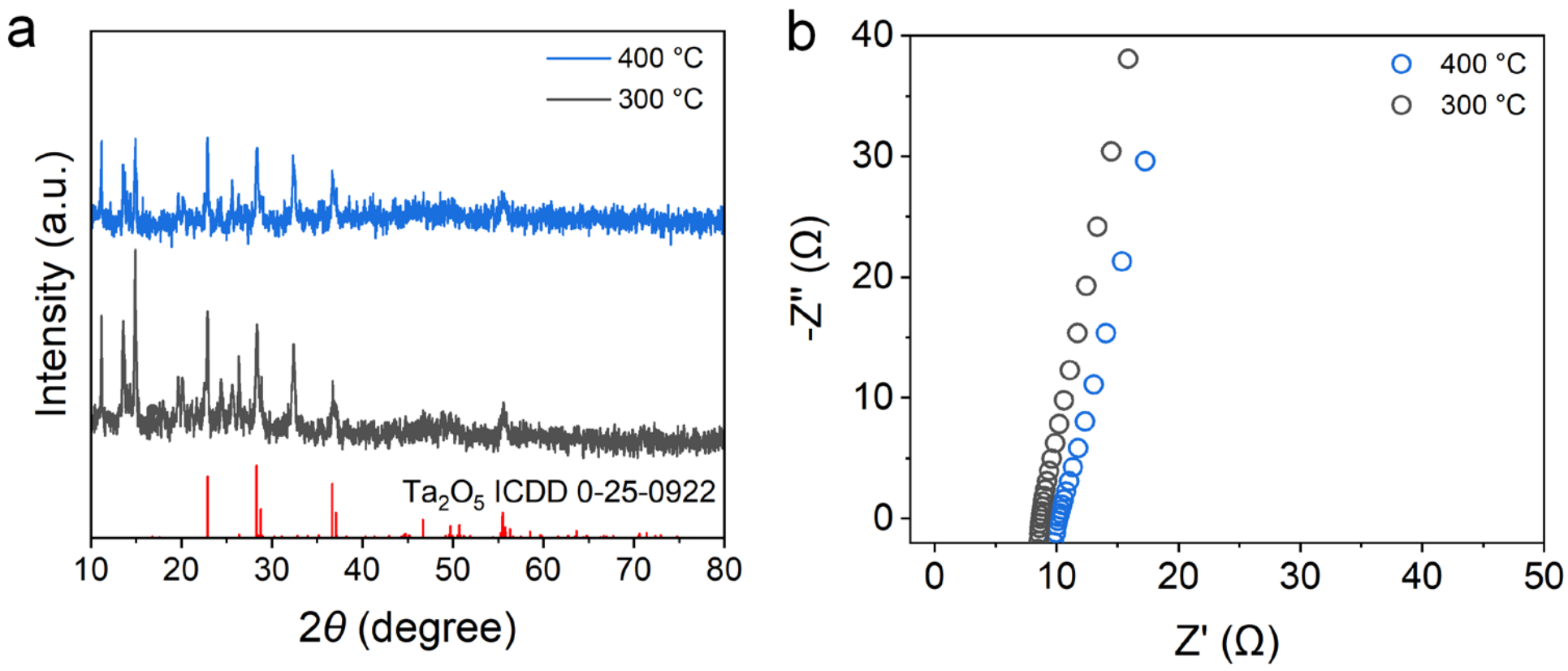

Figure S17. Thermal-treatment control experiment for evaluating the role of the amorphous matrix. (**a**) XRD patterns and (**b**) EIS plots of $Ta_2O_5 \cdot a$-LTOC after thermal treatment at 300 and 400 °C.

After thermal treatment, the amorphous matrix undergoes partial crystallization or structural rearrangement, reducing structural disorder and flexibility. This change disrupts the effective interfacial regions and weakens the connected Li-rich percolation network, thereby lowering the ionic conductivity.

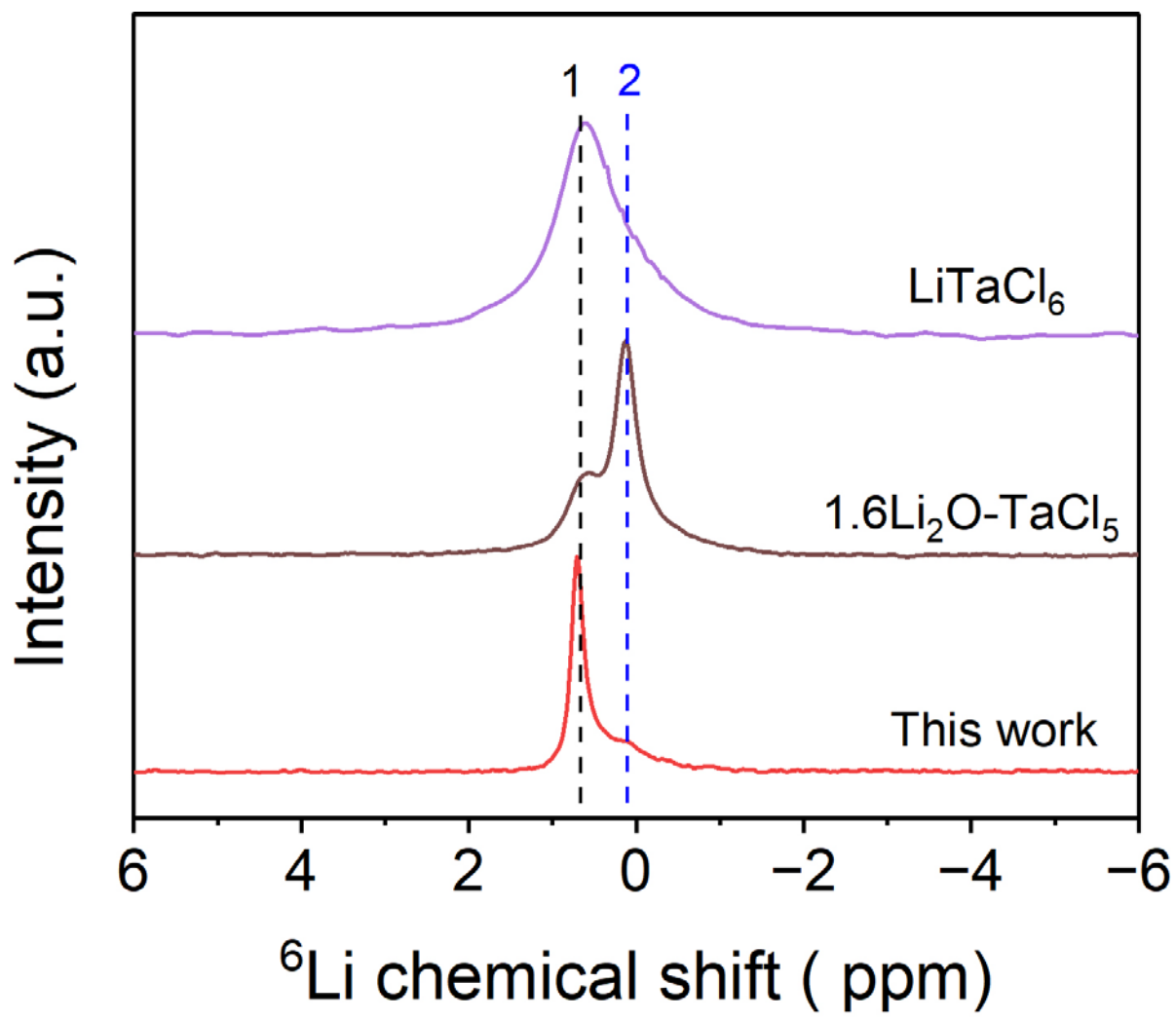

Figure S18. NMR spectra of $LiTaCl_6$, $1.6Li_2O$-$TaCl_5$, and Ta_2O_5·*a*-LTOC in this work.

To further clarify the local Li coordination environments in Ta_2O_5·*a*-LTOC, comparative ^6Li NMR analyses were performed using $LiTaCl_6$ and $1.6Li_2O$-$TaCl_5$ as references[4, 15]. NMR results (Figure S18) show that amorphous $LiTaCl_6$ exhibits a single peak (peak 1), corresponding to a six-coordinated $Li[Cl_6]^-$ environment. $1.6Li_2O$-$TaCl_5$ displays two peaks (peak 1 and peak 2), while peak 2 is attributed to low-coordinated $Li[Cl_{6-x}O_x]^-$ species. In comparison, Ta_2O_5·*a*-LTOC also shows two peaks but with a different intensity ratio. The dominant peak (peak 1) is attributed to the amorphous chloride-rich matrix, whereas the weaker peak (peak 2) indicates the presence of low-coordinated $Li[Cl_{6-x}O_x]^-$ species. These results suggest that the introduction of Ta_2O_5 induces distinct interfacial Li–Cl–O coordination environments.

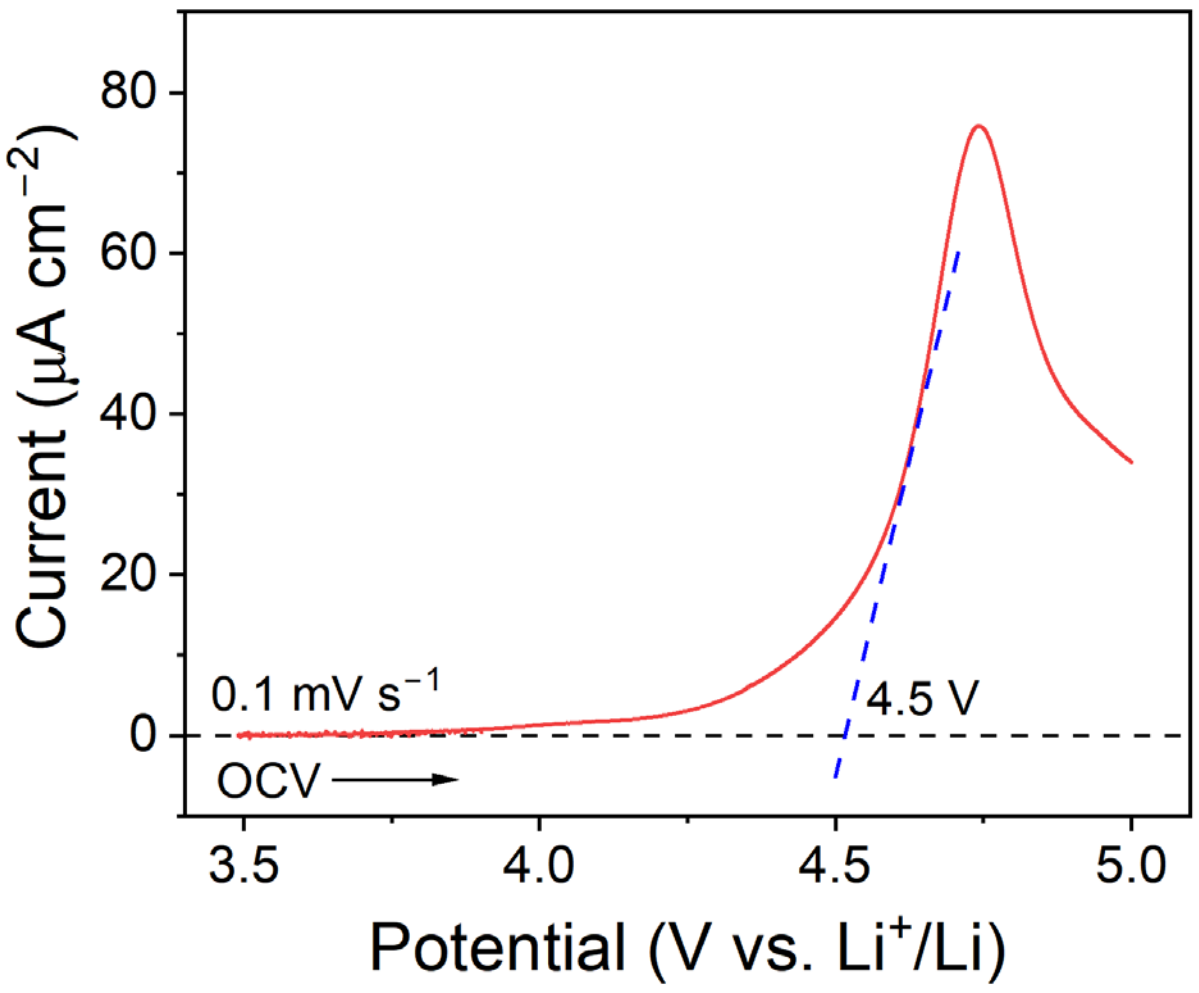

Figure S19. LSV curve of LiIn | LPSC | SSE | SSE+VGCF cell for evaluating the electrochemical stability window of Ta_2O_5·*a*-LTOC.

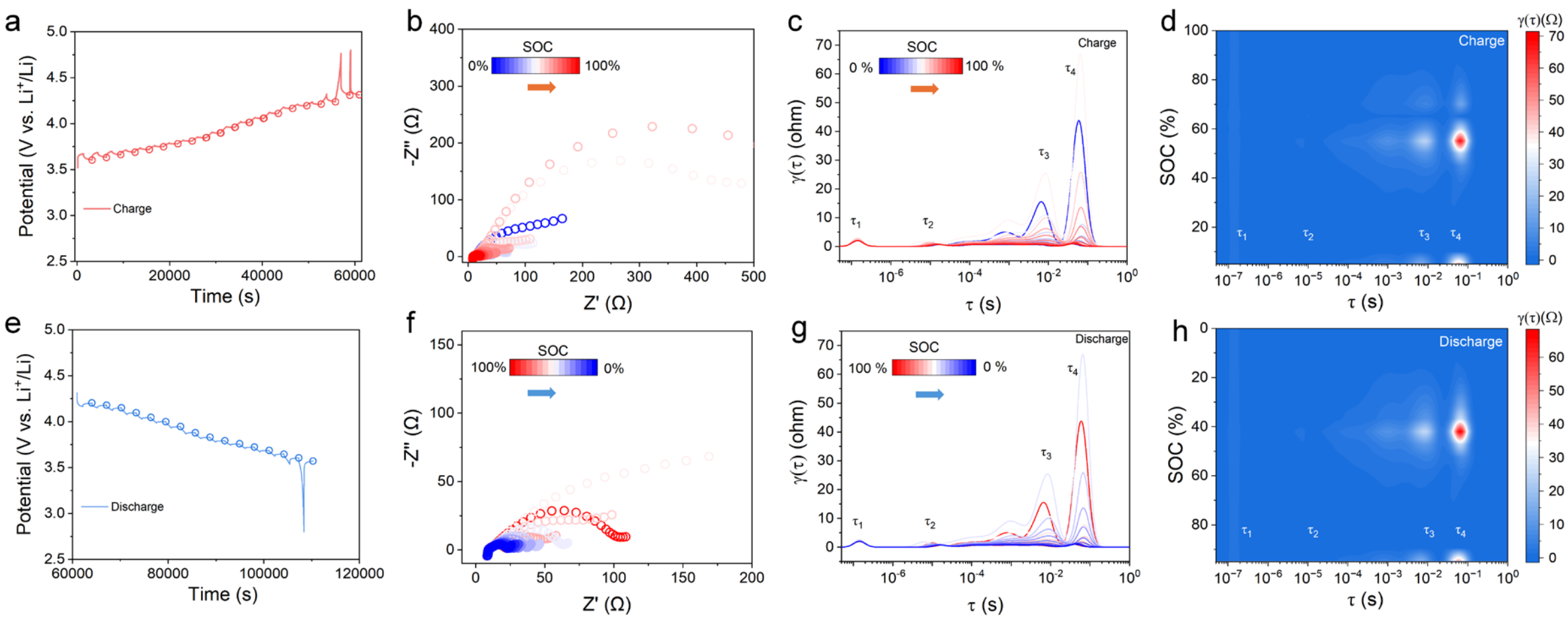

Figure S20. (**a**) Initial charge, and (**e**) discharge curves for Ni90 ASSB at 0.2 C (charged to 4.8 V vs. Li^+/Li). (**b**) EIS plots and corresponding (**c-d**) DRT results at different states of charge (SOC) during charging; (**f**) EIS plots and corresponding (**g-h**) DRT results at different states of charge (SOC) during discharging.

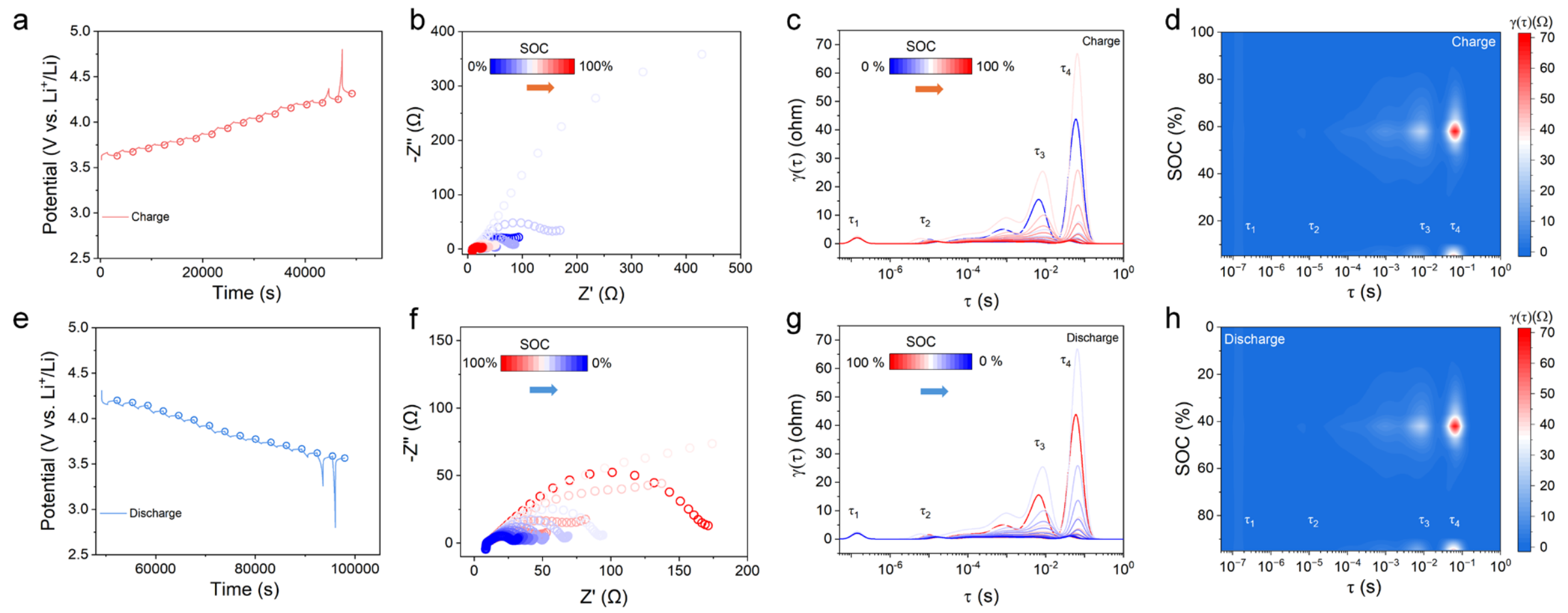

Figure S21. (**a**) Second charge, (**e**) discharge curves for Ni90 ASSB at 0.2 C (charged to 4.8 V vs. Li^+/Li). (**b**) EIS plots and corresponding (**c-d**) DRT results at different states of charge (SOC) during charging; (**f**) EIS plots and corresponding (**g-h**) DRT results at different states of charge (SOC) during discharging.

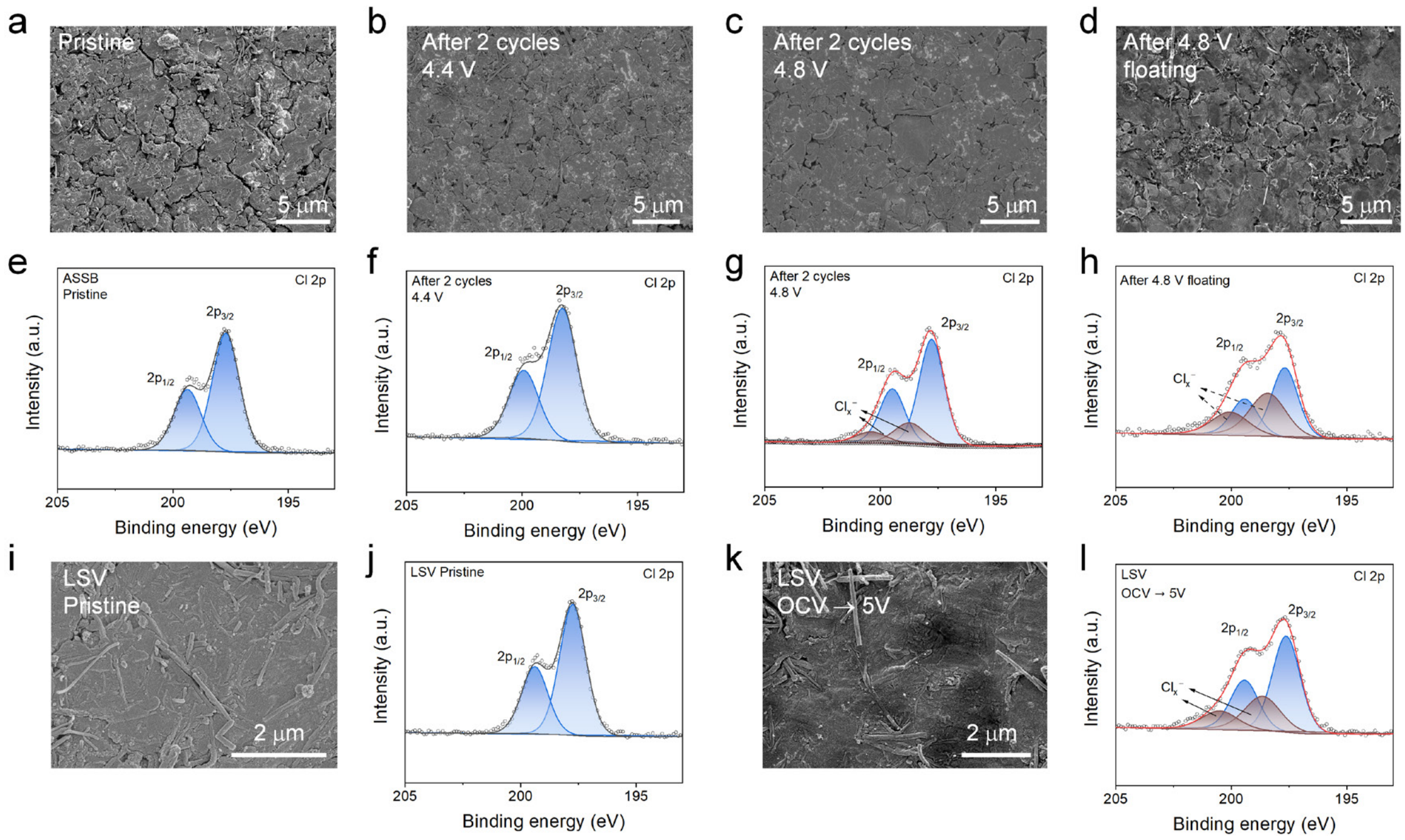

Figure S22. Post-mortem SEM and Cl 2p XPS analyses of Ni90 ASSBs and LSV cells. SEM images of Ni90 ASSBs: (**a**) pristine, (**b**) after 2 cycles at 4.4 V, (**c**) after 2 cycles at 4.8 V, and (**d**) after 4.8 V floating test. Cl 2p XPS spectra of Ni90 ASSBs: (**e**) pristine, (**f**) after 2 cycles at 4.4 V, (**g**) after 2 cycles at 4.8 V, and (**h**) after 4.8 V floating test. LSV cells: (**i**) pristine SEM, (**j**) pristine Cl 2p XPS, (**k**) SEM after scanning from OCV to 5 V, and (**l**) Cl 2p XPS after scanning from OCV to 5 V.

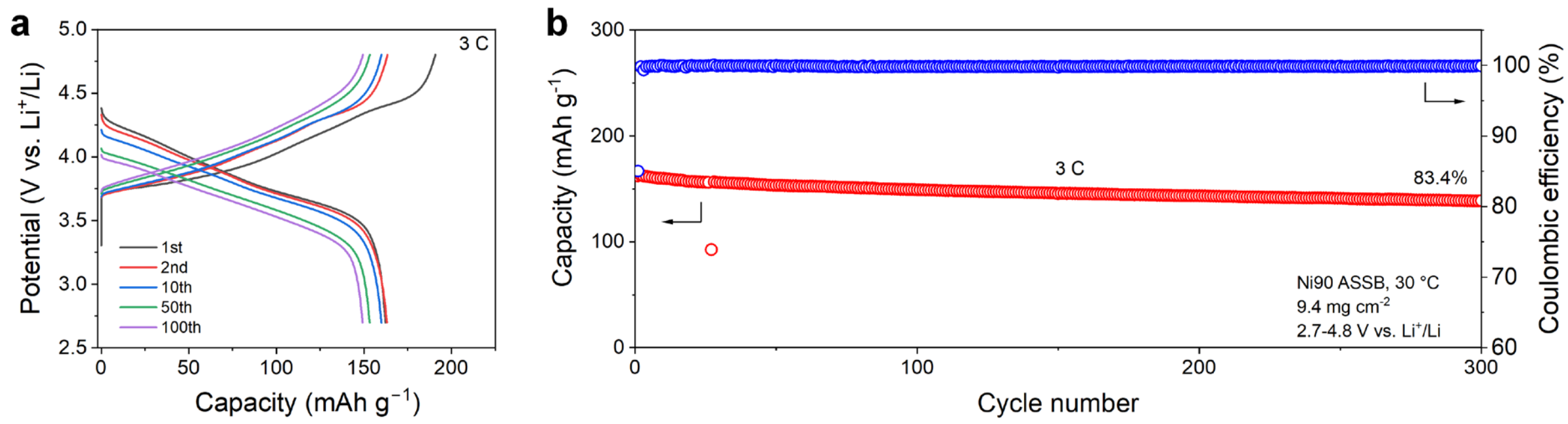

Figure S23. (**a**) Typical charge-discharge voltage profiles and (**b**) corresponding cycling performance of Ni90 ASSB with Ta_2O_5·*a*-LTOC at 3 C in a voltage range of 2.7-4.8 V vs. Li^+/Li.

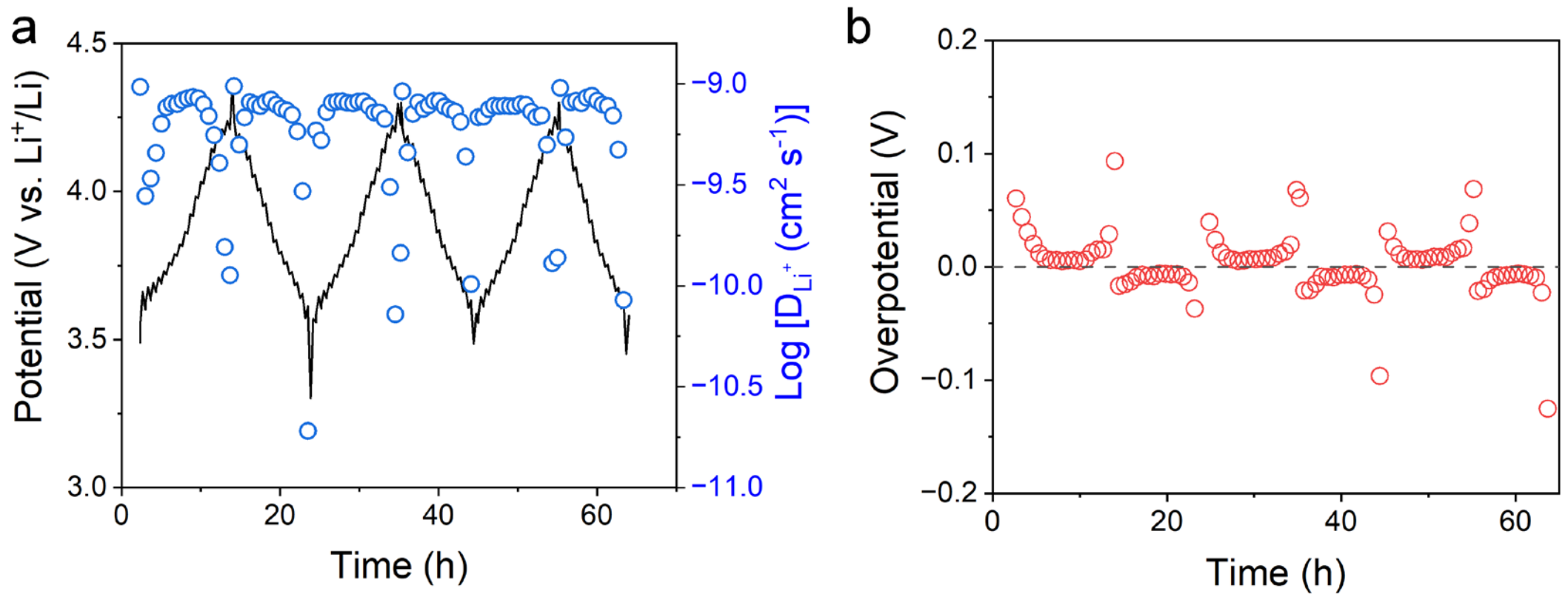

Figure S24. (**a**) GITT curves with Li-ions diffusion coefficients and (**b**) corresponding overpotential of Ni90 ASSB at 0.2 C.

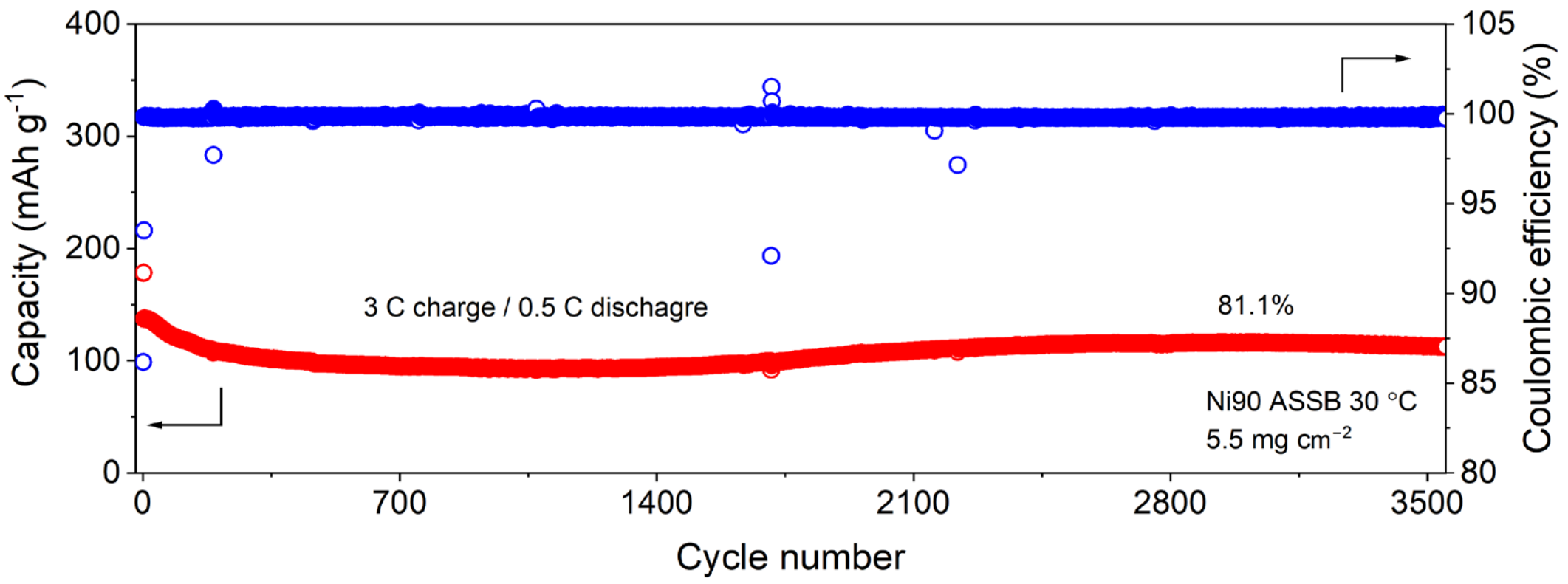

Figure S25. Cycling performance of Ni90 ASSB with $Ta_2O_5 \cdot a$-LTOC under 3 C charge and 0.5 C discharge in a voltage range of 2.8-4.3 V vs. Li^+/Li at 30 °C.

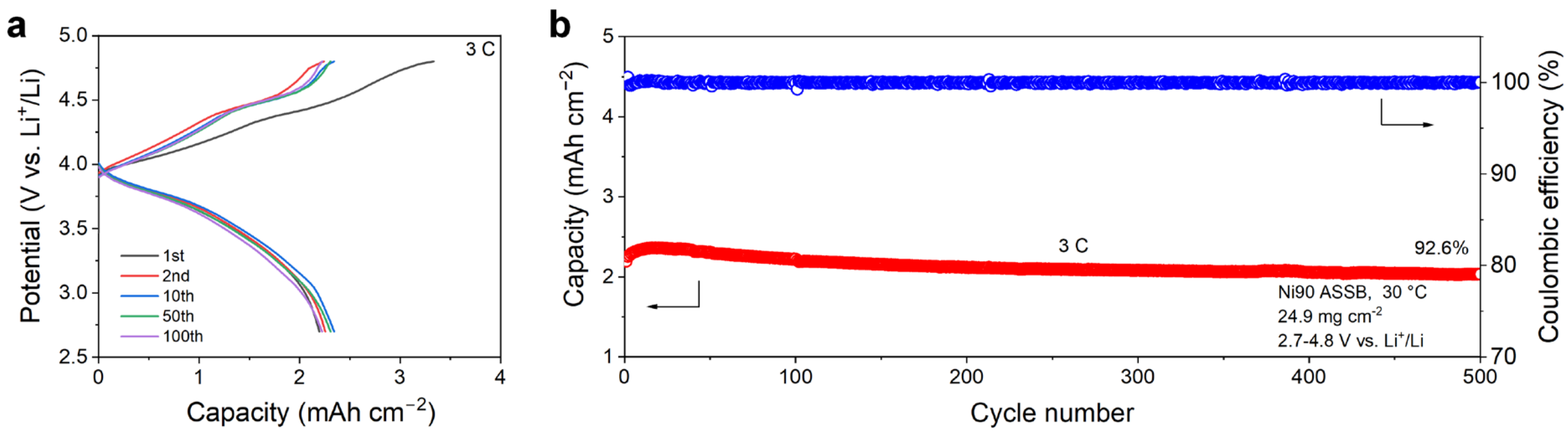

Figure S26. High-loading cycling performance. (**a**) Typical charge-discharge voltage profiles and (**b**) corresponding cycling performance of Ni90 ASSB with $Ta_2O_5 \cdot a$-LTOC at 3 C in a voltage range of 2.7-4.8 V vs. Li^+/Li.

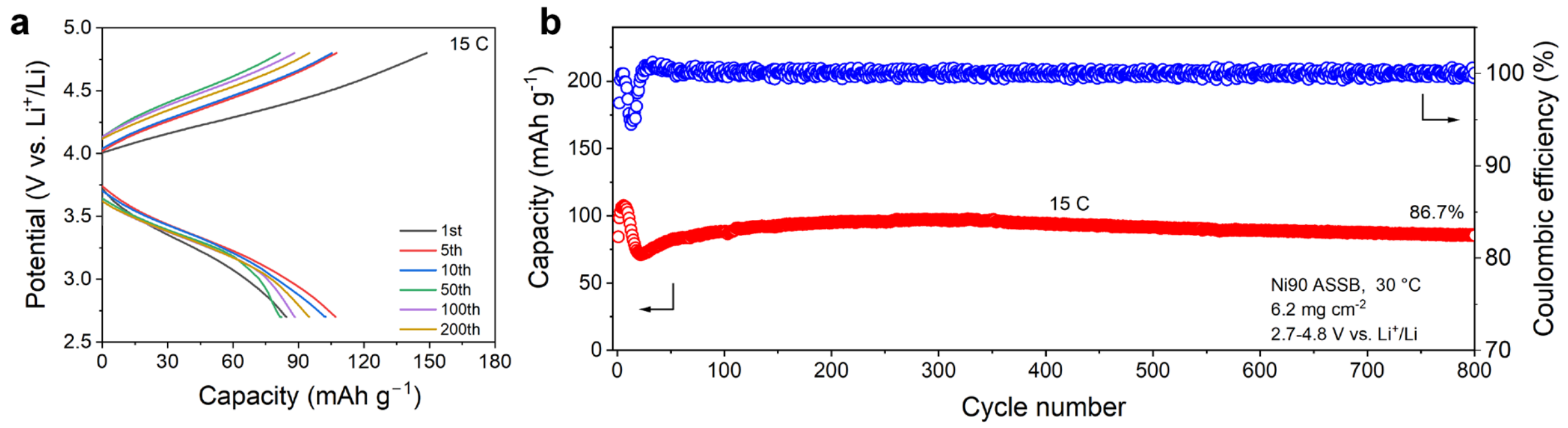

Figure S27. High-rate cycling performance. (**a**) Typical charge-discharge voltage profiles and (**b**) corresponding cycling performance of Ni90 ASSB with $Ta_2O_5 \cdot a$-LTOC at 15 C in a voltage range of 2.7-4.8 V vs. Li^+/Li.

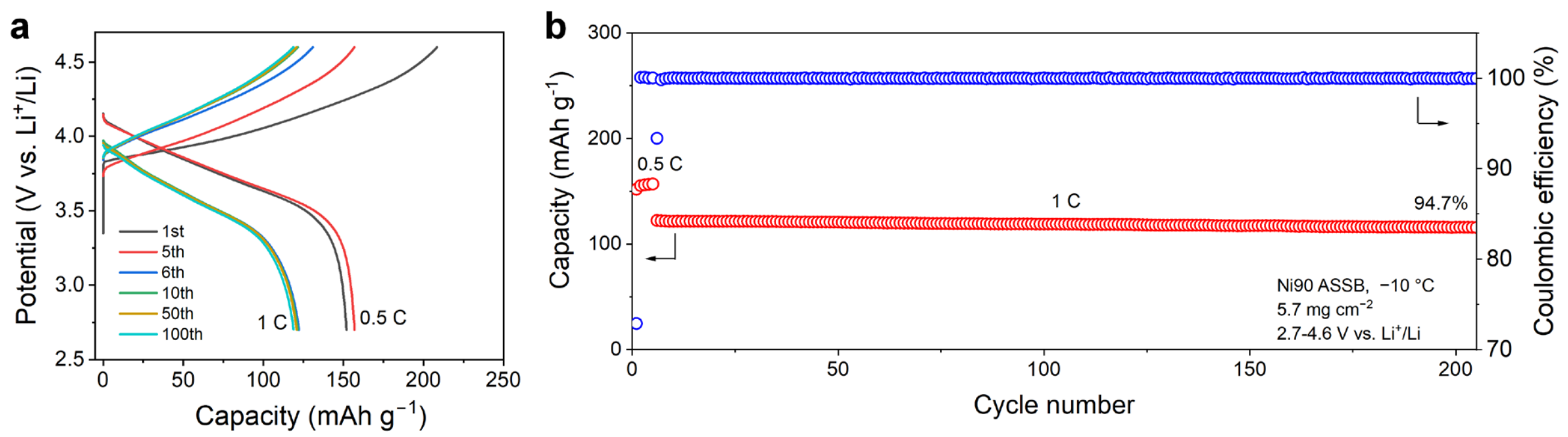

Figure S28. Low-temperature cycling performance. (**a**) Typical charge-discharge voltage profiles and (**b**) corresponding cycling performance of Ni90 ASSB with $Ta_2O_5 \cdot a$-LTOC at −10°C in a voltage range of 2.7-4.6 V vs. Li^+/Li.

Supplemental Tables

Table S1. Ionic conductivities, Li concentration and Li mass fraction of $Li_iTa_{j+2k}O_{5k}Cl_{i+5j}$ SSEs.

**Formula**	**σ (mS cm^{-1})**	**Li concentration (mol L^{-1})**	**Li mass fraction (wt%)**
$Li_{1.63}Ta_3O_{4.5}Cl_{7.63}$	4.4	5.45	1.26
$Li_{1.63}Ta_{2.8}O_4Cl_{7.63}$	5.9	5.73	1.33
$Li_{1.63}Ta_{2.7}O_{3.75}Cl_{7.63}$	8.5	5.88	1.36
$Li_{1.63}Ta_{2.4}O_3Cl_{7.63}$	9.6	6.41	1.48
$Li_{1.63}Ta_{2.2}O_{2.5}Cl_{7.63}$	8	6.79	1.57
$Li_{1.63}Ta_2O_2Cl_{7.63}$	5.5	7.24	1.67
$Li_{1.63}Ta_{1.7}O_{1.25}Cl_{7.63}$	4.6	8.02	1.86
$Li_{1.63}Ta_{1.5}O_{0.75}Cl_{7.63}$	1.3	8.64	2.0
**$Li_{1.6}Ta_{2.4}O_3Cl_{7.6}$**	**15.2**	**6.42**	**1.46**
$Li_{1.56}Ta_{2.4}O_3Cl_{7.56}$	11.8	6.13	1.42
$Li_{1.5}Ta_{2.4}O_3Cl_{7.5}$	10.1	5.91	1.37
$Li_{1.44}Ta_{2.4}O_3Cl_{7.44}$	10	5.71	1.32
$Li_{1.3}Ta_{2.4}O_3Cl_{7.3}$	8.2	5.2	1.2
$Li_{1.2}Ta_{2.4}O_3Cl_{7.2}$	6.4	4.83	1.12
$Li_{1.1}Ta_{2.4}O_3Cl_{7.1}$	6.2	4.45	1.03
$Li_1Ta_{2.4}O_3Cl_7$	4.2	4.07	0.94
$Li_{0.75}Ta_{2.4}O_3Cl_{6.75}$	2.2	3.06	0.72
**$Li_{0.5}Ta_{2.4}O_3Cl_{6.5}$**	**0.5**	**2.09**	**0.48**
$Li_{1.63}Ta_3O_5Cl_{6.63}$	5	5.62	1.30
$Li_{1.6}Ta_{2.8}O_{3.5}Cl_{8.6}$	11.85	5.43	1.26
$Li_{1.6}Ta_{2.46}O_3Cl_{7.9}$	7.8	5.91	1.42
$Li_{1.6}Ta_{2.34}O_3Cl_{7.3}$	10.2	6.46	1.50

Table S2. Curve fit parameters [a] for Ta L_3-edge EXAFS for $Ta_2O_5 \cdot a$-LTOC.

Shell	CN [b]	R (Å) [c]	Debye–Waller factor ($Å^2$) [d]
Ta-O1	1.4±0.4 (2.0 in Ta_2O_5)	1.63±0.02 (1.938 in Ta_2O_5)	0.0061±0.0004
Ta-O2	3.6±0.4 (3.0 in Ta_2O_5)	1.83±0.02 (2.001 in Ta_2O_5)	0.0061±0.0004
Ta-Cl1	1.0±0.2 (2.0 in $TaCl_5$)	2.28±0.02 (2.277 in $TaCl_5$)	0.0082±0.0004
Ta-Cl2	2.0±0.2 (2.0 in $TaCl_5$)	2.39±0.02 (2.323 in $TaCl_5$)	0.0082±0.0004
Ta-Cl3	1.0±0.2 (2.0 in $TaCl_5$)	2.70±0.02 (2.569 in $TaCl_5$)	0.0082±0.0004
Ta-Ta1	2.7±0.2 (2.0 in Ta_2O_5)	3.37±0.02 (3.457 in Ta_2O_5)	0.0055±0.0004
Ta-Ta2	3.5±0.2 (2.0 in Ta_2O_5)	3.54±0.02 (3.712 in Ta_2O_5)	0.0055±0.0004
Ta-O-Ta	2.4±0.2 (4.0 in Ta_2O_5)	3.59±0.02 (3.869 in Ta_2O_5)	0.0065±0.0004
Ta-Ta3	1.5±0.2 (2.0 in Ta_2O_5)	4.03±0.02 (3.875 in Ta_2O_5)	0.0055±0.0004

(a) Data ranges: $3 \leq k \leq 12$ $Å^{-1}$, $1 \leq R \leq 4$ Å, (b) CN = coordination number; (c) R = distance between absorber and backscatter atoms; (d) The numbers in the brackets correspond to the theoretical CN and R values, Ta-O paths from the crystal structures of Ta_2O_5 (mp-1104837); Ta-Cl paths from the crystal structure of $TaCl_5$ (mp-29831). R factor for this fit is 1.12%.

Table S3. Comparison of E_a, pre-exponential factor (σ_0), Li content, ^{7}Li NMR T_1 and T_2 relaxation times (Figure S13), ionic conductivity, and conductivity-to-Li-content ratio ($\sigma/n_{Li,wt}$) for representative SSEs. The pre-exponential factor was extracted from the linear fitting of ln(σT) versus 1000/T.

SSEs	E_a (eV)	T_1 (s)	T_2 (ms)	σ_0 (S K cm^{-1})	$n_{Li,wt}$ (wt%)	σ (mS cm^{-1})	$\sigma/n_{Li,wt}$
$Li_{1.6}Ta_{2.4}O_3Cl_{7.6}$ (Ta_2O_5·*a*-LTOC)	0.24	0.88	10.21	4.3×10^4	1.46	15.2	10.4
$Li_{1.3}Ta_{2.4}O_3Cl_{7.3}$	0.26	0.81	17.95	5.75×10^4	1.2	8.2	6.8
$Li_{1.63}Ta_{2.4}O_3Cl_{7.63}$	0.27	-	-	7.31×10^4	1.48	9.6	6.49
$LiTaCl_6$	0.28	0.30	1.13	3.90×10^4	1.73	3	1.73

Table S4. Comparison of ionic conductivity as a function of Li mass fraction among typical oxide, sulfide, and halide SSEs.

SSEs	σ (mS cm^{-1})	Li mass fraction (wt%)	Reference
**$Ta_2O_5 \cdot a$-LTOC**	**15.2**	**1.46**	**This work**
Li_3YCl_6	0.51	6.46	[16]
Li_3YBr_6	0.72	3.53	[16]
Li_3InCl_6	1.49	5.98	[17]
Li_3ScCl_6	3.02	7.48	[18]
$Li_{10}GeP_2S_{12}$	12	11.79	[19]
$Li_{0.34}La_{0.51}TiO_{2.94}$	1	1.40	[20]
$Li_2Sc_{2/3}Cl_4$	1.5	7.48	[21]
$Li_7La_3Zr_2O_{12}$	0.3	5.79	[22]
Li_3HoCl_6	0.1	5.23	[23]
Li_3ErCl_6	0.33	5.19	[24]
Li_2ZrCl_6	0.81	4.37	[25]
$Li_2In_{1/3}Sc_{1/3}Cl_4$	2.0	6.64	[26]
$Li_{3.2}Ta_5O_{1.6}Cl_5$	6.6	1.97	[4]
$LiTaOCl_4$	12.4	2.01	[27]
$LiNbOCl_4$	10.4	2.69	[27]
$Li_{2.5}Lu_{0.5}Zr_{0.5}Cl_6$	1.5	4.78	[28]
A-$LiTaCl_6 \cdot LiCl$	7.16	3.13	[15]
Li_6PS_5Cl	1.9	15.52	[29]
Li_6PS_5Br	6.8	13.31	[29]
$Li_{1.3}Al_{0.3}Ti_{1.7}(PO_4)_3$	0.1	2.35	[30]
$Li_{10}SnP_2S_{12}$	4	10.93	[31]
$0.5Li_2SO_4$-$ZrCl_4$	1.5	2.4	[32]

Table S5. Ionic conductivities of MO_y·LiM'OCl with different compositions.

**Precursors**	**Formula**	**σ (mS cm^{-1})**
$LiCl$-$TaCl_5$-Bi_2O_3	$Li_{1.6}Ta_{1.2}Bi_{1.2}O_{1.8}Cl_{7.6}$	0.32
$LiCl$-$TaCl_5$-Sb_2O_3	$Li_{1.6}Ta_{1.2}Sb_{1.2}O_{1.8}Cl_{7.6}$	0.15
$LiCl$-$TaCl_5$-ZrO_2	$Li_{1.6}Ta_{1.2}Zr_{1.2}O_{2.4}Cl_{7.6}$	4.4
$LiCl$-$TaCl_5$-SnO_2	$Li_{1.6}Ta_{1.2}Sn_{1.2}O_{1.2}Cl_{7.6}$	2.5
$LiCl$-$TaCl_5$-ZnO	$Li_{1.6}Ta_{1.2}Zn_{1.2}O_{1.2}Cl_{7.6}$	1.2
$LiCl$-$TaCl_5$-MoO_3	$Li_{1.6}Ta_{1.2}Mo_{1.2}O_{3.6}Cl_{7.6}$	0.8
$LiCl$-$TaCl_5$-Al_2O_3	$Li_{1.6}Ta_{1.2}Al_{1.2}O_{1.8}Cl_{7.6}$	3.3
$LiCl$-$TaCl_5$-VO_2	$Li_{1.6}Ta_{1.2}V_{1.2}O_{3.2}Cl_6$	0.6
$LiCl$-$TaCl_5$-Nb_2O_5	$Li_{1.6}Ta_{1.2}Nb_{1.2}O_3Cl_{7.6}$	5.5
$LiCl$-$TaCl_5$-SiO_2	$Li_{1.5}Ta_{1.2}Si_{1.2}O_{2.4}Cl_6$	3.7
$LiBr$-$TaCl_5$-Ta_2O_5	$Li_{1.6}Ta_{2.4}O_3Cl_6Br_{1.6}$	3.6
$NaCl$-$TaCl_5$-Ta_2O_5	$Na_{1.56}Ta_{2.4}O_3Cl_{7.56}$	3.5
$LiCl$-$NbCl_5$-Ta_2O_5	$Li_{1.6}Ta_{1.2}Nb_{1.2}O_3Cl_{7.6}$	1.4
Li_2O-$ZrCl_4$-Ta_2O_5	$Li_{1.6}Ta_{1.2}Zr_{1.2}O_{3.8}Cl_{4.8}$	2.3
Li_2O-$ZrCl_4$-ZrO_2	$Li_{1.6}Zr_{2.4}O_{3.2}Cl_{4.8}$	2.2
Li_2O-$ZrCl_4$-Al_2O_3	$Li_{1.6}Zr_{1.2}Al_{1.2}O_{2.6}Cl_{4.8}$	1.38
Li_2O-$ZrCl_4$-SiO_2	$Li_{1.6}Zr_{1.2}Si_{1.2}O_{3.2}Cl_{4.8}$	1.36

References:

1. Ravel B, Newville M. ATHENA, ARTEMIS, HEPHAESTUS: data analysis for X-ray absorption spectroscopy using IFEFFIT. *J Synchrotron Radiat* 2005; **12**: 537–541.
2. Bauhofer W, Kovacs JZ. A review and analysis of electrical percolation in carbon nanotube polymer composites. *Compos Sci Technol* 2009; **69**: 1486–1498.
3. Bunde A, Dieterich W, Roman E. Dispersed ionic conductors and percolation theory. *Phys Rev Lett* 1985; **55**: 5–8.
4. Zhang S, Zhao F, Chen J *et al.* A family of oxychloride amorphous solid electrolytes for long-cycling all-solid-state lithium batteries. *Nat Commun* 2023; **14**: 3780.
5. Lei M, Li B, Liu H *et al.* Dynamic monkey bar mechanism of superionic Li-ion transport in $LiTaCl_6$. *Angew Chem Int Ed* 2024; **63**: e202315628.
6. Liu Y, Wang S, Nolan AM *et al.* Tailoring the cation lattice for chloride lithium-ion conductors. *Adv Energy Mater* 2020; **10**: 2002356.
7. Zhao F, Zhang S, Wang S *et al.* Revealing unprecedented cathode interface behavior in all-solid-state batteries with oxychloride solid electrolytes. *Energy Environ Sci* 2024; **17**: 4055-4063.
8. Babushkina OB, Ekres S. Spectroscopic study of the electrochemical behaviour of tantalum(V) chloride and oxochloride species in 1-butyl-1-methylpyrrolidinium chloride. *Electrochim Acta* 2010; **56**: 867-877.
9. Mal'chik AG, Kuznetsova SA, Kryuchkova SO *et al.* Sol–gel synthesis of Ta_2O_5–SiO_2 composites from tantalum(V) chloride and tetraethyl orthosilicate in ethanol. *Inorg Mater* 2017; **53**: 994-1003.
10. Wang Y, Qu H, Liu B *et al.* Self-organized hetero-nanodomains actuating super Li^+ conduction in glass ceramics. *Nat Commun* 2023; **14**: 669.
11. Guo C, Shen Y, Mao P *et al.* Grafting of Lithiophilic and Electron-Blocking Interlayer for Garnet-Based Solid-State Li Metal Batteries via One-Step Anhydrous Poly-Phosphoric Acid Post-Treatment. *Adv Funct Mater* 2023; **33**: 2213443.
12. Zhan X, Pang X, Mao F *et al.* Interfacial Reconstruction Unlocks Inherent Ionic Conductivity of Li-La-Zr-Ta-O Garnet in Organic Polymer Electrolyte for Durable Room-Temperature All-Solid-State Batteries. *Adv Energy Mater* 2024; **14**: 2402509.
13. Bloembergen N, Purcell EM, Pound RV. Relaxation Effects in Nuclear Magnetic Resonance Absorption. *Phys Rev* 1948; **73**: 679-712.
14. Uitz M, Epp V, Bottke P *et al.* Ion dynamics in solid electrolytes for lithium batteries. *J Electroceram* 2017; **38**: 142-156.
15. Li F, Cheng X, Lu G *et al.* Amorphous chloride solid electrolytes with high Li-ion conductivity for stable cycling of all-solid-state high-nickel cathodes. *J Am Chem Soc* 2023; **145**: 27774–27787.
16. Asano T, Sakai A, Ouchi S *et al.* Solid halide electrolytes with high lithium-ion conductivity for application in 4 V class bulk-type all-solid-state batteries. *Adv Mater* 2018; **30**: 1803075.
17. Li X, Liang J, Luo J *et al.* Air-stable Li_3InCl_6 electrolyte with high voltage compatibility for all-solid-state batteries. *Energy Environ Sci* 2019; **12**: 2665-2671.
18. Liang J, Li X, Wang S *et al.* Site-occupation-tuned superionic Li_xScCl_{3+x} halide solid

electrolytes for all-solid-state batteries. *J Am Chem Soc* 2020; **142**: 7012-7022.

19. Kamaya N, Homma K, Yamakawa Y *et al.* A lithium superionic conductor. *Nat Mater* 2011; **10**: 682-686.
20. Inaguma Y, Liquan C, Itoh M *et al.* High ionic conductivity in lithium lanthanum titanate. *Solid State Commun* 1993; **86**: 689-693.
21. Zhou L, Kwok CY, Shyamsunder A *et al.* A new halospinel superionic conductor for high-voltage all solid state lithium batteries. *Energy Environ Sci* 2020; **13**: 2056-2063.
22. Murugan R, Thangadurai V, Weppner W. Fast Lithium Ion Conduction in Garnet-Type $Li_7La_3Zr_2O_{12}$. *Angew Chem Int Ed* 2007; **46**: 7778-7781.
23. Zhou L, Zuo T, Li C *et al.* $Li_{3-x}Zr_x(Ho/Lu)_{1-x}Cl_6$ Solid Electrolytes Enable Ultrahigh-Loading Solid-State Batteries with a Prelithiated Si Anode. *ACS Energy Lett* 2023; **8**: 3102-3111.
24. Muy S, Voss J, Schlem R *et al.* High-Throughput Screening of Solid-State Li-Ion Conductors Using Lattice-Dynamics Descriptors. *iScience* 2019; **16**: 270-282.
25. Wang K, Ren Q, Gu Z *et al.* A cost-effective and humidity-tolerant chloride solid electrolyte for lithium batteries. *Nat Commun* 2021; **12**: 4410.
26. Zhou L, Zuo T-T, Kwok CY *et al.* High areal capacity, long cycle life 4 V ceramic all-solid-state Li-ion batteries enabled by chloride solid electrolytes. *Nat Energy* 2022; **7**: 83-93.
27. Tanaka Y, Ueno K, Mizuno K *et al.* New Oxyhalide Solid Electrolytes with High Lithium Ionic Conductivity >10 mS cm^{-1} for All-Solid-State Batteries. *Angew Chem Int Ed* 2023; **62**: e202217581.
28. Wang Ch, Wang S, Liu Xd *et al.* New insights into aliovalent substituted halide solid electrolytes for cobalt-free all-solid state batteries. *Energy Environ Sci* 2023; **16**: 5136-5143.
29. Rao RP, Adams S. Studies of lithium argyrodite solid electrolytes for all-solid-state batteries. *Phys Status Solidi A* 2011; **208**: 1804-1807.
30. Mertens A, Yu S, Schön N *et al.* Superionic bulk conductivity in $Li_{1.3}Al_{0.3}Ti_{1.7}(PO_4)_3$ solid electrolyte. *Solid State Ion* 2017; **309**: 180-186.
31. Bron P, Johansson S, Zick K *et al.* $Li_{10}SnP_2S_{12}$: An Affordable Lithium Superionic Conductor. *J Am Chem Soc* 2013; **135**: 15694-15697.
32. Tang W, Wang F, Liang S *et al.* Polyanion-stabilized amorphous halide electrolytes with low lithium content for all-solid-state lithium batteries. *Nat Commun* 2026.
